# Dynamics of hybrid switching DS-I-A epidemic model

**DOI:** 10.1038/s41598-017-11901-x

**Published:** 2017-09-26

**Authors:** Songnan Liu, Daqing Jiang, Xiaojie Xu, Tasawar Hayat, Bashir Ahmad

**Affiliations:** 1 College of Science, China University of Petroleum (East China), Qingdao, 266580 China; 2 Nonlinear Analysis and Applied Mathematics (NAAM)-Research Group, Department of Mathematics, King Abdulaziz University, Jeddah, Saudi Arabia; 3 Department of Mathematics, Quaid-I-Azam University 45320, Islamabad, 44000 Pakistan

## Abstract

In this paper, we investigate a stochastic hybrid switching DS-I-A epidemic model. The extinction and the prevalence of the disease are discussed, and so, the threshold is given. Furthermore, the sufficient conditions for the existence of positive recurrence of the solutions are established by stochastic Lyapunov functions. At last, some examples and simulations are provided to illustrate our results.

## Introduction

Human immunodeficiency virus (HIV) infection is characterized by three different phases, namely the primary infection, clinically asymptomatic stage (chronic infection), and acquired immunodeficiency syndrome (AIDS) or drug therapy. Basic developed techniques for measuring HIV RNA levels are allowing researchers to develop a picture of HIV infection patterns. HIV–1 RNA levels in plasma and serum become extremely high during the 1–2 weeks of acute primary infection, before there was a detectable immune response^1,2^. These levels are higher than at any other time during infection. Acute primary infection is followed by a chronic phase. During the chronic phase, HIV RNA levels drop several orders of magnitude and remain ‘nearly constant’ for years^3–5^, where ‘nearly constant’ includes fluctuations that are less than an order of magnitude up and down for about 90% of the cohort and less than a factor of 100 for the remaining^4^. Fluctuations may be caused by transient illnesses and vaccinations. Successful therapy causes a drop in the viral load to a new level that is maintained until viral resistance develops^6^. Viral levels differ by many orders of magnitude between individuals. Those people with high viral loads in the chronic phase tend to progress rapidly to AIDS, whereas those with very low loads tend to be slow or nonprogressors^4,5,7,8^. During late chronic infection, there is a small increase in HIV-1 RNA levels, at most tenfold, in many individuals^3^.

Mathematical modeling is useful for understanding the spread of HIV/AIDS. Thus various models have been developed to describe the spread of this disease according to its characteristics, see refs^9–13^. A simple homogeneous AIDS model has given by the following system of ODEs^13^. The model classifies the sexually active population into three classes that are: susceptibles, infectives and AIDS cases, with population numbers in each class denoted as functions of time by *S*(*t*), *I*(*t*) and *A*(*t*) respectively. Sexually mature susceptibles *S*(*t*), contain sexually mature people in the population who have had no contact with the virus. This compartment increases through maturation of individuals into sexually mature age group and decreases by contagion going to the next compartment and natural death. Sexually mature infectives *I*(*t*), contains sexually mature individuals who are infected with the virus but have not yet developed AIDS symptoms. The number of people in this group would decrease through natural death and development to AIDS after a certain stay in this class (develop symptomatic AIDS). AIDS cases *A*(*t*), are those individuals who have developed fully symptomatic AIDS and exhibit specific clinical features and this class would decrease by natural death and AIDS-related death. The population is assumed to be uniform and homogeneously mixing. The total adult population and sexually interacting adult population are denoted by *N*(*t*) = *S*(*t*) + *I*(*t*). A recruitment-death demographic structure is assumed. Individuals enter the susceptible class at a constant rate *μS* ^0^ > 0. The natural death rate is assumed to be proportional to the population number in each class, with rate constant *μ* > 0. The model assumes a constant rate *γ* > 0 of development to AIDS. In addition, there is an AIDS-related death in the AIDS class which is assumed to be proportional to the population number in that class, with rate constant *δ* > 0 which is the sum of natural mortality rate and mortality due to illness. In most epidemiological models, bilinear incidence rate is frequently used. The incidence implies that the contact number between susceptible individuals and infected individuals is proportional to susceptible individuals. But it is more realistic that the rate of infection depends on the transmission probability per contact of individuals and the proportion of individuals. Thus the model assumes standard incidence of the form $\frac{\beta \alpha S(t)\,I(t)}{N(t)}$ where *β* > 0 is the probability of being infected from a new sexual partner, *α* > 0 is the is the susceptibility of susceptible individuals and *βα* > 0 becomes the average number of effective contacts of one infective individual per unit time:

1.1 $$\{\begin{array}{rcl}\frac{dS(t)}{dt} & = & \mu ({S}^{0}-S(t))-\frac{\beta \alpha S(t)\,I(t)}{N(t)},\\ \frac{dI(t)}{dt} & = & \frac{\beta \alpha S(t)\,I(t)}{N(t)}-(\mu +\gamma )\,I(t),\\ \frac{dA(t)}{dt} & = & \gamma I(t)-\delta A(t),\end{array}$$

where the parameters *μ*, *S* ^0^, *α*, *γ* and $\delta \in {{\mathbb{R}}}_{+}$. (1.1) assume a homogeneous susceptible population such that there is one group of susceptible individuals, the mean number of contacts, the mean transmission probability, and the mean duration of infection can be defined so that the reproductive number can be always given as the product of these three means. However genetic evidence now exists that individuals may be exhibiting differential susceptibility to the infection. Host genetic factors play a major role in determining the susceptibility to infectious diseases. Further studies are needed to determine the hosts differential susceptibility to various disease as well as its implications to public health. In ref.^13^, James M. Hyman and Jia Li, formulated compartmental differential susceptibility (DS) susceptible-infective-AIDS (SIA) models by dividing the susceptible population into multiple subgroups according to the susceptibility of individuals in each group. They derived an explicit formula for the reproductive number of infection for each model. They further proved that the infection-free equilibrium and endemic equilibria of each model were globally asymptotically stable. In this paper, we consider a simple differential susceptibility (DS) model in which the infected population is homogeneous^13^:

1.2 $$\{\begin{array}{rcl}\frac{d{S}_{k}(t)}{dt} & = & \mu ({S}_{k}^{0}-{S}_{k}(t))-\frac{\beta {\alpha }_{k}{S}_{k}(t)\,I(t)}{N(t)},\quad 1\le k\le n,\\ \frac{dI(t)}{dt} & = & \sum _{k=1}^{n}\frac{\beta {\alpha }_{k}{S}_{k}(t)\,I(t)}{N(t)}-(\mu +\gamma )\,I(t),\\ \frac{dA(t)}{dt} & = & \gamma I(t)-\delta A(t),\end{array}$$

in which $N(t)={\sum }_{k=1}^{n}\,{S}_{k}(t)+I(t)$, ${S}_{i}(t)\,(i=1,2,\ldots ,n)$ denote the *n* individuals susceptible to infection subgroups. Hence, the individuals in each group have homogeneous susceptibility, but the susceptibilities of individuals from different groups are distinct. The susceptibles are distributed into *n* susceptible subgroups based on their inherent susceptibilities. This is done in such a way that the input flow into group *S*_*k*_ is $\mu {S}_{k}^{0}$ (*k* = 1, 2, …, *n*); *α*_*k*_(*k* = 1, 2, …, *n*) the susceptibility of susceptible individuals in subgroup *I* and $\frac{\beta {\alpha }_{k}I(t)\,{S}_{k}(t)}{N(t)}$ the standard incidence ratio of susceptible subgroups *S*_*k*_. Since the dynamics of group *A* has no effect on the disease transmission dynamics, thus we only consider

1.3 $$\{\begin{array}{rcl}\frac{d{S}_{k}(t)}{dt} & = & \mu ({S}_{k}^{0}-{S}_{k}(t))-\frac{\beta {\alpha }_{k}{S}_{k}(t)\,I(t)}{N(t)},\quad 1\le k\le n,\\ \frac{dI(t)}{dt} & = & \sum _{k=1}^{n}\frac{\beta {\alpha }_{k}{S}_{k}(t)\,I(t)}{N(t)}-(\mu +\gamma )\,I(t),\end{array}$$

The threshold conditions can be calculated which determine whether an infectious disease will spread in susceptible population when the disease is introduced into the crowed, according to research the disease free equilibrium ${E}_{0}({S}_{1}^{0},{S}_{2}^{0},\ldots ,{S}_{n}^{0},\mathrm{0)}$ of system (1.3) in ref.^14^.

And reproductive number obtained in ref.^13^

1.4 $${R}_{0}=\frac{\beta \,{\sum }_{k=1}^{n}{\alpha }_{k}{S}_{k}^{0}}{(\mu +\gamma )\,{\sum }_{k=1}^{n}{S}_{k}^{0}}.$$

If *R*_0_ < 1, *E*_0_ is local asymptotic stabile and disease extinct. When *R*_0_ > 1, then *E*_0_ is unstable and the disease will persistent existence (see ref.^13^). The effective contact rate of infected individual in subgroup *S*_*k*_(*k* = 1, 2, …, *n*) is *α*_*k*_ *β*(*k* = 1, 2, …, *n*). So for initial time $({S}_{i}={S}_{i}^{0})$, the average effective contact rate of infected individual in subgroup *S*_*k*_(*k* = 1, 2, …, *n*) is $\frac{\beta {\sum }_{k=1}^{n}\,{\alpha }_{k}{S}_{k}^{0}}{{\sum }_{k=1}^{n}\,{S}_{k}^{0}}$. $\frac{1}{\mu +\gamma }$ the average disease period of infected individuals. So *R*_0_ is basic reproductive number.

It is well recognized fact that real life is full of randomness and stochasticity. Hence the epidemic models are always affected by the environmental noise (in cite refs^15–22^). In refs^23–28^, the stochastic models may be more convenient epidemic models in many situations^29^. Have previously used the technique of parameter perturbation to examine the effect of environmental stochasticity in a model of AIDS and condom use. They found that the introduction of stochastic noise changes the basic reproduction number of the disease and can stabilize an otherwise unstable system. Thus in this paper, we first introduce white noise to consider the small perturbation in environment. To establish the stochastic differential equation (SDE) model, we naturally use the equation in the form of differential

1.5 $$d{S}_{k}(t)=[\mu ({S}_{k}^{0}-{S}_{k}(t))-\frac{\beta {\alpha }_{k}{S}_{k}(t)\,I(t)}{N(t)}]\,dt,\quad 1\le k\le n.$$

Here [*t*, *t* + Δ*t*) is a small time interval and *d*⋅ for the small change. For example *dS*_*k*_(*t*) = *S*_*k*_(*t* + *dt*) − *S*_*k*_(*t*), 1 ≤ *k* ≤ *n* and the change *dS*_*k*_(*t*) is described by (1.5). Consider the effective contact rate constant of infected individual *βα*_*k*_, 1 ≤ *k* ≤ *n* in the deterministic model. The total number of newly increased *I* in the small interval [*t*, *t* + *dt*) is

$$\sum _{k=1}^{n}\,\frac{\beta {\alpha }_{k}{S}_{k}(t)\,I(t)}{N(t)}dt.$$

Now suppose that some stochastic environment factors act simultaneously on each subgroups in the disease. In this case, *βα*_*k*_, 1 ≤ *k* ≤ *n* changes to a random variable $\tilde{\beta {\alpha }_{k}}$, 1 ≤ *k* ≤ *n*. More precisely

$$\tilde{\beta {\alpha }_{k}}dt=\beta {\alpha }_{k}dt+{\sigma }_{k}d{B}_{k}(t)\quad 1\le k\le n.$$

Here *dB*_*k*_(*t*) = *B*_*k*_(*t* + *dt*) − *B*_*k*_(*t*) (*k* = 1, 2, …, *n*) is the increment of a standard Brownian motion. And *B*_*k*_(*t*) (*k* = 1, 2, …, *n*) are independent standard Brownian motions with *B*_*k*_(0) = 0 (*k* = 1, 2, …, *n*) and ${\sigma }_{k}^{2} > 0$ $(k=1,2,\ldots ,n)$ denote the intensities of the white noise. Thus the number of newly increasing *I* that each subgroups *S*_*k*_, 1 ≤ *k* ≤ *n* infected in [*t*, *t* + *dt*] is normally distributed with mean *βα*_*k*_ *dt* and variance ${\sigma }_{k}^{2}dt$, where *k* = 1, 2, …, *n*.

Therefore we replace *βα*_*k*_ *dt* in equation (1.5) by $\tilde{\beta {\alpha }_{k}}dt=\beta {\alpha }_{k}dt+{\sigma }_{k}dB(t)$ to get

$$d{S}_{k}(t)=[\mu ({S}_{k}^{0}-{S}_{k}(t))-\tfrac{\beta {\alpha }_{k}{S}_{k}(t)\,I(t)}{N(t)}]\,dt-{\sigma }_{k}\tfrac{{S}_{k}(t)\,I(t)}{N(t)}d{B}_{k}(t),\quad 1\le k\le n.$$

Note that *βα*_*i*_ *dt* now denotes the mean of the stochastic number of *S*_*i*_ infected in the infinitesimally small time interval [*t*, *t* + *dt*). Similarly, the first equation of (1.3) becomes another SDE. That is, the deterministic infectious diseases model (1.3) becomes the $It\,\hat{o}$’s SDE

1.6 $$\{\begin{array}{rcl}d{S}_{k}(t) & = & [\mu ({S}_{k}^{0}-{S}_{k}(t))-\tfrac{\beta {\alpha }_{k}{S}_{k}(t)\,I(t)}{N(t)}]\,dt-{\sigma }_{k}\tfrac{{S}_{k}(t)\,I(t)}{N(t)}d{B}_{k}(t),\quad 1\le k\le n.\\ dI(t) & = & [\sum _{k=1}^{n}\tfrac{\beta {\alpha }_{k}{S}_{k}(t)\,I(t)}{N(t)}-(\mu +\gamma )\,I(t)]\,dt+\sum _{k=1}^{n}{\sigma }_{k}\tfrac{{S}_{k}(t)\,I(t)}{N(t)}d{B}_{k}(t),\end{array}$$

Other parameters are the same as in system (1.3). Besides white noise, epidemic models may be disturbed by telegraph noise which makes population systems switch from one regime to another. Let us now take a further step by considering another type of environmental noise, namely, color noise, say telegraph noise (see refs^30^ and^31^). The telegraph noise can be illustrated as a switching between two or more regimes of environment, which differs by factors such as nutrition or as rain falls^32–35^. For example, the growth rate for some fish in dry season will be much different from it in rainy season. Telegraph noise can also be illustrated as a switching between different environments, which differ by factors such as climatic characteristics or socio-cultural factors. The latter may cause the disease to spread faster or slower. Frequently, the switching among different environments is memoryless and the waiting time for the next switch is exponentially distributed^36^. Therefore the regime switching can be modeled by a continuous time finite-state Markov chain (*ξ*(*t*))_*t*≥0_ with values in a finite state space ${\mathcal M} =\{1,2,\ldots ,L\}$. In this paper, we consider the HIV disease spread between environmental regimes. Because the HIV epidemic model may be influenced by different social cultures, we also introduce the telegraph noise to consider HIV disease spread between different social cultures that is the large disturbance in environment. That is following stochastic DS-I-A model disturbed by white and telephone noises.

1.7 $$\{\begin{array}{rcl}d{S}_{k}(t) & = & [\mu (l)\,({S}_{k}^{0}(l)-{S}_{k}(t))-\tfrac{\beta (l){\alpha }_{k}(l){S}_{k}(t)\,I(t)}{N(t)}]\,dt-{\sigma }_{k}(l)\tfrac{{S}_{k}(t)\,I(t)}{N(t)}d{B}_{k}(t),\\  &  & \quad 1\le k\le n.\\ dI(t) & = & [\sum _{k=1}^{n}\tfrac{\beta (l){\alpha }_{k}(l){S}_{k}(t)\,I(t)}{N(t)}-(\mu (l)+\gamma (l))\,I(t)]\,dt+\sum _{k=1}^{n}{\sigma }_{k}(l)\tfrac{{S}_{k}(t)\,I(t)}{N(t)}d{B}_{k}(t),\end{array}$$

The switching between these *L* regimes is governed by a Markov chain on the state space ${\mathcal M} =\{1,2,\ldots ,L\}$. The DS-I-A systems under regime switching can therefore be described by the following stochastic model (SDE):

18 $$\{\begin{array}{rcl}d{S}_{k}(t) & = & [\mu (\xi (t))\,({S}_{k}^{0}\xi (t)-{S}_{k}(t))-\tfrac{\beta (\xi (t)){\alpha }_{k}(\xi (t)){S}_{k}(t)\,I(t)}{N(t)}]\,dt-{\sigma }_{k}(\xi (t))\tfrac{{S}_{k}(t)\,I(t)}{N(t)}d{B}_{k}(t),\\  &  & \quad 1\le k\le n.\\ dI(t) & = & [\sum _{k=1}^{n}\tfrac{\beta (\xi (t)){\alpha }_{k}(\xi (t)){S}_{k}(t)\,I(t)}{N(t)}-(\mu (\xi (t))+\gamma (\xi (t)))\,I(t)]\,dt+\sum _{k=1}^{n}{\sigma }_{k}(\xi (t))\tfrac{{S}_{k}(t)\,I(t)}{N(t)}d{B}_{k}(t),\end{array}$$

where *ξ*(*t*) is a continuous time Markov chain with values in finite state space ${\mathcal M} =\{1,2,\ldots ,L\}$, the parameters $\mu (l)$, ${S}_{k}^{0}(l)$, ${\sigma }_{k}(l)$, $\beta (l)$, ${\alpha }_{k}(l)$, $\gamma (l)$, $k=1,2,\ldots ,n$, are all positive constants for each $l\in  {\mathcal M}$. This system is operated as follows: If *ξ*(1) = *l*_1_, the system obeys systems (1.7) with *l* = *l*_1_ till time *τ*_1_ when the Markov chain jumps to *l*_2_ from *l*_1_; the systems will then obey (1.7) with *l* = *l*_2_ from *τ*_1_ till *τ*_2_ when the Markov chain jumps to *l*_3_ from *l*_2_. The system will continue to switch as long as the Markov chain jumps. In other words, the SDE (1.6) can be regarded as (1.7) switching from one to another according to the law of the Markov Chain. Each of (1.7) $l\in  {\mathcal M}$ is hence called a subsystem of the SDE (1.6). We aim to investigate the positive recurrence and extinction. Since system (1.6) is perturbed by both white and telegraph noises, the existence of positive recurrence of the solutions is an important issue. However, to the best of our knowledge, there has been no result related this. In this paper, we attempt to do some work in this field to fill the gap. The theory we used is developed by Zhu and Yin ref.^37^. The key difficulty is how to construct a suitable Lyapunov function and a bounded domain. So one of the main aim of this paper is to establish sufficient conditions for the existence of positive recurrence of the solutions to system (1.6).

This paper is organized as follows. In Section 2, we present some preliminaries that will be used in our following analysis. In Section 3, we show that there exists a unique global positive solution of system (1.8). In Section 4, we establish sufficient conditions for extinction of disease. The condition for the disease being persistent is given in Sections 5. In Section 6, we show the existence of positive recurrence.

## Preliminaries

Throughout this paper, unless otherwise specified, let $({\rm{\Omega }}, {\mathcal F} ,{\{{ {\mathcal F} }_{t}\}}_{t\ge 0},P)$ be a complete probability space with a filtration ${\{{ {\mathcal F} }_{t}\}}_{t\ge 0}$ satisfying the usual conditions(i.e. it is right continuous and ${ {\mathcal F} }_{0}$ contains all *P*-null sets). Denote

$${{\mathbb{R}}}_{+}^{n+1}=\{x\in {{\mathbb{R}}}^{n+1}:{x}_{i} > 0\,{\rm{for}}\,{\rm{all}}\,1\le i\le n+1\}.$$

We consider the general *n* + 1-dimensional stochastic differential equation

2.1 $$dx(t)=f(x(t),t)\,dt+g(x(t),t)\,dB(t),\,{\rm{for}}\,t\ge {t}_{0}$$

with initial value $x({t}_{0})={x}_{0}\in {{\mathbb{R}}}^{n+1}$, where *B*(*t*) denotes *n* + 1-dimensional standard Brownian motions defined on the above probability space.

Define the differential operator ${\mathcal L}$ associated with Eq. (2.1) by

$$ {\mathcal L} =\frac{\partial }{\partial t}+{\rm{\Sigma }}\,{f}_{i}(x,t)\frac{\partial }{\partial {x}_{i}}+\frac{1}{2}{\rm{\Sigma }}\,{[{g}^{T}(x,t)g(x,t)]}_{ij}\frac{{\partial }^{2}}{\partial {x}_{i}\partial {x}_{j}}.$$

If ${\mathcal L}$ acts on a function *V* ∈ *C* ^2,1^ $({{\mathbb{R}}}^{n}\times {{\mathbb{R}}}_{+};{{\mathbb{R}}}_{+})$, then

$$ {\mathcal L} V(x,t)={V}_{t}(x,t)+{V}_{x}(x,t)\,f(x,t)+\frac{1}{2}trac[{g}^{T}(x,t)\,{V}_{xx}(x,t)\,g(x,t)]$$

where ${V}_{t}=\frac{\partial V}{\partial t}$, ${V}_{x}=(\frac{\partial V}{\partial {x}_{1}},\ldots ,\frac{\partial V}{\partial {x}_{n+1}})$ and ${V}_{xx}={(\frac{{\partial }^{2}V}{\partial {x}_{i}\partial {x}_{j}})}_{n+1\times n+1}$. By It $\hat{o}$’s formula, if *x*(*t*) is a solution of Eq. (2.1), then

$$dV(x(t),t)= {\mathcal L} V(x(t),t)\,dt+{V}_{x}(x(t),t)\,g(x(t),t)\,dB(t).$$

In Eq. (2.1), we assume that *f*(0, *t*) = 0 and *g*(0, *t*) = 0 for all *t* ≥ *t*_0_. So *x*(*t*) ≡ 0 is a solution of Eq. (2.1), called the trivial solution or equilibrium position.

By the definition of stochastic differential, the equation (2.1) is equivalent to the following stochastic integral equation

2.2 $$x(t)={x}_{0}+{\int }_{{t}_{0}}^{t}f(x(s),s)\,ds+\sum _{r=1}^{n+1}{\int }_{{t}_{0}}^{t}{g}_{r}(x(s),s)\,d{B}_{r}(s),\,for\,t\ge {t}_{0}.$$

For any vector *g* = (*g*(1), …, *g*(*L*)), set $\hat{g}={{\rm{\min }}}_{k\in  {\mathcal M} }\,\{g(k)\}$ and $\breve{g}={{\rm{\max }}}_{k\in  {\mathcal M} }\,\{g(k)\}$. Suppose the generator Γ = (*γ*_*ij*_)_*L*×*L*_ of the Markov chain is given by

$$P\{\xi (t+\delta )=j|\xi (t)=i\}=\{\begin{array}{ll}{\gamma }_{ij}\delta +o(\delta ), & {\rm{if}}\,i\ne j,\\ 1+{\gamma }_{ij}\delta +o(\delta ), & {\rm{if}}\,i=j,\end{array}$$

where *δ* > 0, *γ*_*ij*_ ≥ 0 for any *i* ≠ *j* is the transition rate from *i* to *j* if *i* ≠ *j* while ${\sum }_{j=1}^{L}\,{\gamma }_{ij}=0$. In this paper, we assume *γ*_*ij*_ > 0, for any *i* ≠ *j*. Assume further that Markov chain *ξ*(*t*) is irreducible and has a unique stationary distribution *π* = {*π*_1_, *π*_2_, …, *π*_*L*_} which can be determined by equation

2.3 $$\pi {\rm{\Gamma }}=0,$$

subject to

$$\sum _{h=1}^{L}\,{\pi }_{h}=1,\,{\rm{and}}\,{\pi }_{h} > 0,\,\forall h\in  {\mathcal M} .$$

We assume that Brownian motion and Markov chain are independent.

Let (*X*(*t*), *ξ*(*t*)) be the diffusion process described by the following equation:

2.4 $$dX(t)=b(X(t),\xi (t))\,dt+\sigma (X(t),\xi (t))\,dB(t),\,X(0)={x}_{0},\xi (0)=\xi ,$$

where $b(\cdot ,\cdot ):{{\mathbb{R}}}^{n}\times  {\mathcal M} \to {{\mathbb{R}}}^{n},\sigma (\cdot ,\cdot ):{{\mathbb{R}}}^{n}\times  {\mathcal M} \to {{\mathbb{R}}}^{n\times n}$, and *D*(*x*, *l*) = *σ*(*x*, *l*)*σ* ^*T*^ (*x*, *l*) = (*d*_*ij*_(*x*, *l*)). For each $l\in  {\mathcal M}$, let *V*(·, *l*) be any twice continuously differentiable function, the operator ${\mathcal L}$ can be defined by

$$ {\mathcal L} V(x,l)=\sum _{i=1}^{n}\,{b}_{i}(x,l)\frac{\partial V(x,l)}{\partial {x}_{i}}+\frac{1}{2}\,\sum _{i,j=1}^{n}\,{d}_{ij}(x,l)\frac{{\partial }^{2}V(x,l)}{\partial {x}_{i}\partial {x}_{j}}+\sum _{m=1}^{L}\,{\gamma }_{lm}V(x,l).$$

## Existence and Uniqueness of Positive Solution

In this section we first show that the solution of system (1.8) is positive and global. To get a unique global (i.e. no explosion in a finite time) solution for any initial value, the coefficients of the equation are required to satisfy the linear growth condition and the local lipschitz condition. However, the coefficients of system (1.8) do not satisfy the linear growth condition, as the item $\frac{\beta {\alpha }_{k}{S}_{k}(t)\,I(t)}{N(t)}$ is nonlinear. So the solution of system (1.8) may explode to infinity in a finite time. In this section, we show that the solution of system (1.8) is positive and global by using the Lyapunov analysis method.

### **Theorem 3**.**1**

*There is a unique positive solution X*(*t*) = (*S*_1_(*t*), *S*_2_(*t*), …, *S*_*n*_(*t*), *I*(*t*)) *of system* (*1*.*8*) *on t* ≥ 0 *for any initial value* $({S}_{1}\mathrm{(0)},{S}_{2}\mathrm{(0)},\ldots ,{S}_{n}\mathrm{(0)},I\mathrm{(0}))\in {{\mathbb{R}}}_{+}^{n+1}$, *and the solution will remain in* ${{\mathbb{R}}}_{+}^{n+1}$ *with probability 1*, *namely*, $({S}_{1}(t),{S}_{2}(t),\ldots ,{S}_{n}(t),I(t))\in {{\mathbb{R}}}_{+}^{n+1}$ *for all t* ≥ 0.

*Proof*. Since the coefficients of the equation are locally Lipschitz continuous for given initial value $({S}_{1}^{0},{S}_{2}^{0},\ldots ,{S}_{n}^{0},{I}_{0})\in {R}_{+}^{n+1}$. There is a unique local solution (*S*_1_(*t*), *S*_2_(*t*), …, *S*_*n*_(*t*), *I*(*t*)) on *t* ∈ [0,*τ*_*e*_], where *τ*_*e*_ is the explosion time^18^. To show the solution is global, we only need to verify that *τ*_*e*_ = ∞ a.s. Let *m*_0_ ≥ 0 be sufficiently large so that every component of *X*(0) lies within the interval [1/*m*_0_, *m*_0_]. For each *m* ≥ *m*_0_, we define the stopping time

$$\begin{array}{rcl}{\tau }_{m} & = & {\rm{\inf }}\,\{t\in [0,{\tau }_{e}]:\,{\rm{\min }}\,\{{S}_{1}(t),{S}_{2}(t),\ldots ,{S}_{n}(t),I(t)\}\\  & \le  & \frac{1}{m}\,or\,{\rm{\max }}\,\{{S}_{1}(t),{S}_{2}(t),\ldots ,{S}_{n}(t),I(t)\}\ge m\}\end{array}$$

where we set ${\rm{\inf }}\,\,\varphi =\infty$ (as usual $\varphi$ denotes the empty set) throughout the paper. According to the definition, *τ*_*m*_ is increasing when *m* → ∞. Set ${\tau }_{\infty }=\mathop{\mathrm{lim}}\limits_{m\to \infty }\,{\tau }_{m}$, then *τ*_∞_ ≤ *τ*_*e*_ a.s. In the following, we need to prove that *τ*_∞_ = ∞ a.s., then *τ*_*e*_ = ∞ and $({S}_{1}(t),{S}_{2}(t),\ldots ,{S}_{n}(t),I(t))\in {{\mathbb{R}}}_{+}^{n+1}$ a.s. for all *t* ≥ 0. In other words, to complete the proof all we need to show is that *τ*_∞_ = ∞ a.s. If this assertion is violated then there exists a pair of constants *T* > 0 and *ε* ∈ (0, 1) such that

$$P\{{\tau }_{\infty }\le T\} > \varepsilon .$$

Hence there is an integer *m*_1_ ≥ *m*_0_ such that

$$P\{{\tau }_{\infty }\le T\}\ge \varepsilon ,\,for\,all\,m\ge {m}_{1}.$$

For *t* ≤ *τ*_*m*_, we can see, for each *m*,

$$\begin{array}{rcl}d(\sum _{k\mathrm{=1}}^{n}{S}_{k}+I) & = & [\mu (\xi (t))\sum _{k=1}^{n}\,({S}_{k}^{0}(\xi (t))-{S}_{k})-(\mu (\xi (t))+\gamma (\xi (t)))I]\,dt\\  & = & [\mu (\xi (t))\sum _{k=1}^{n}\,{S}_{k}^{0}(\xi (t))-\mu (\xi (t))\,(\sum _{k=1}^{n}\,{S}_{k}+I)-\gamma (\xi (t))I]\,dt\\  & \le  & \mu (\xi (t))\sum _{k=1}^{n}\,{S}_{k}^{0}(\xi (t))dt-\mu (\xi (t))\,(\sum _{k=1}^{n}\,{S}_{k}+I)\,dt.\end{array}$$

Therefore

$$\sum _{k=1}^{n}\,{S}_{k}(t)+I(t)\le \{\begin{array}{ll}\sum _{k=1}^{n}\,{{\check{S}}}_{k}^{0}, & {\rm{if}}\,\sum _{k\mathrm{=1}}^{n}\,{S}_{k}\mathrm{(0)}+I\mathrm{(0)} < \sum _{k=1}^{n}\,{{\check{S}}}_{k}^{0},\\ \sum _{k=1}^{n}\,{S}_{k}\mathrm{(0)}+I\mathrm{(0),} & {\rm{if}}\,\sum _{k=1}^{n}\,{S}_{k}\mathrm{(0)}+I\mathrm{(0)}\ge \sum _{k=1}^{n}\,{{\check{S}}}_{k}^{0}.\end{array}$$

Let $C\,:=\,{\rm{\max }}\,\{{\sum }_{k=1}^{n}\,{{\check{S}}}_{k}^{0},{\sum }_{k=1}^{n}\,{S}_{k}\mathrm{(0)}+I\mathrm{(0)}\}$. Define a *C* ^2^-function $V:{{\mathbb{R}}}_{+}^{n+1}\to {{\mathbb{R}}}_{+}$ by

$$V({S}_{1},{S}_{2},\ldots ,{S}_{n},I,l)=\sum _{k=1}^{n}\,({S}_{k}-1-\,\mathrm{ln}\,{S}_{k})+(I-1-\,\mathrm{ln}\,I).$$

The non-negativity of this function can be see from $u-1-\,\mathrm{log}\,u\ge 0$, $\forall u > 0$. Let *m* ≥ *m*_0_ and *T* > 0 be arbitrary then by It $\hat{o}$’s formula one obtains

3.1 $$\begin{array}{rcl} {\mathcal L} V & = & \sum _{k=1}^{n}(1-\frac{1}{{S}_{k}})\,[\mu (l)\,({S}_{k}^{0}(l)-{S}_{k})-\frac{\beta (l){\alpha }_{k}(l)\,{S}_{k}I}{N}]+(1-\frac{1}{I})\\  &  & \times [\sum _{k=1}^{n}\frac{\beta (l){\alpha }_{k}(l)\,{S}_{k}I}{N}-(\mu (l)+\gamma (l))I]\\  &  & +\sum _{k=1}^{n}\frac{{\sigma }_{k}^{2}(l)}{2}\frac{{I}^{2}}{{N}^{2}}+\sum _{k=1}^{n}\frac{{\sigma }_{k}^{2}(l)}{2}\frac{{S}_{k}^{2}}{{N}^{2}}\\  & = & \mu (l)\,\sum _{k=1}^{n}{S}_{k}^{0}(l)-\mu (l)\,(\sum _{k=1}^{n}{S}_{k}+I)\\  &  & -\gamma (l)I-\mu (l)\,\sum _{k=1}^{n}\frac{{S}_{k}^{0}(l)}{{S}_{k}}+(n+1)\,\mu (l)+\gamma (l)\\  &  & +\frac{\beta (l)I}{N}\,\sum _{k=1}^{n}{\alpha }_{k}(l)-\sum _{k=1}^{n}\frac{\beta (l){\alpha }_{k}(l){S}_{k}}{N}\\  &  & +\sum _{k=1}^{n}\frac{{\sigma }_{k}^{2}(l)}{2}\frac{{I}^{2}}{{N}^{2}}+\sum _{k=1}^{n}\frac{{\sigma }_{k}^{2}(l)}{2}\frac{{S}_{k}^{2}}{{N}^{2}}\\  &  <  & \mathop{\mu }\limits^{\breve{}}\,\sum _{k=1}^{n}{\mathop{{S}}\limits^{\breve{}}}_{k}^{0}+(n+1)\,\mathop{\mu }\limits^{\breve{}}+\mathop{\gamma }\limits^{\breve{}}+\mathop{\beta }\limits^{\breve{}}\,\sum _{k=1}^{n}{\mathop{\alpha }\limits^{\breve{}}}_{k}+\sum _{k=1}^{n}{\mathop{\sigma }\limits^{\breve{}}}_{k}^{2}:=M,\end{array}$$

where *M* is a positive constant which is independent of *S*_1_, *S*_2_, …, *S*_*n*_, *I* and *t*. The remainder of the proof follows that in ref.^38^.

### Remark 3.1

*From Theorem 3*.*1 there is a unique global solution* $({S}_{1}(t),{S}_{2}(t),\ldots ,{S}_{n}(t),I(t))\in {{\mathbb{R}}}_{+}^{n+1}$ *almost surely of system* (*1.8*), *for any initial value* $({S}_{1}\mathrm{(0)},{S}_{2}\mathrm{(0)},\ldots ,{S}_{n}\mathrm{(0),}\,I\mathrm{(0))}\in {{\mathbb{R}}}_{+}^{n+1}$. *Hence*

$$d(\sum _{k=1}^{n}\,{S}_{k}(t)+I(t))\le \mu (\xi (t))\,\sum _{k=1}^{n}\,{S}_{k}^{0}(\xi (t))dt-\mu (\xi (t))\,(\sum _{k=1}^{n}\,{S}_{k}+I)dt,$$

*and*

$$\sum _{k=1}^{n}\,{S}_{k}(t)+I(t)\le \sum _{k=1}^{n}\,{{\check{S}}}_{k}^{0}+{e}^{-{\int }_{0}^{t}\mu (\xi (s))ds}\,[\sum _{k=1}^{n}\,{S}_{k}\mathrm{(0)}+I\mathrm{(0)}-\sum _{k=1}^{n}\,{{\check{S}}}_{k}^{0}].$$

*If* ${\sum }_{k=1}^{n}\,{S}_{k}\mathrm{(0)}+I\mathrm{(0)} < {\sum }_{k=1}^{n}\,{{\check{S}}}_{k}^{0}$, *then* ${\sum }_{k=1}^{n}\,{S}_{k}(t)+I(t) < {\sum }_{k=1}^{n}\,{{\check{S}}}_{k}^{0}\,a.s.$ *Thus the region*

$${{\rm{\Gamma }}}^{\ast }=\{({S}_{1},{S}_{2},\ldots ,{S}_{n},I)\in {{\mathbb{R}}}_{+}^{n+1},\,\sum _{k=1}^{n}\,{S}_{k}(t)+I(t) < \sum _{k=1}^{n}\,{{\check{S}}}_{k}^{0}\}$$

*is a positively invariant set of system* (1.8).

## Extinction

The other main concern in epidemiology is how we can regulate the disease dynamics so that the disease will be eradicated in a long term. In this section, we shall give a sharp result of the extinction of disease in the stochastic model (1.8).

### Theorem 4.1

If following conditions satisfied:

(i) ${\bar{R}}_{0}^{\ast }:=\frac{{\sum }_{k=1}^{n}\,{\sum }_{l=1}^{L}\,{\pi }_{l}\frac{{\beta }^{2}(l){\alpha }_{k}^{2}(l)}{2{\sigma }_{k}^{2}(l)}}{{\sum }_{l=1}^{L}\,{\pi }_{l}(\mu (l)+\gamma (l))} < 1$, *then the disease I*(*t*) *will die out exponentially with probability one*, *i*.*e*.,
  $$\mathop{\mathrm{lim}\,{\rm{\sup }}}\limits_{t\to \infty }\,\frac{\mathrm{ln}\,I(t)}{t}\le \sum _{l=1}^{L}\,{\pi }_{l}(\mu (l)+\gamma (l))\,({\bar{R}}_{0}^{\ast }-\mathrm{1)} < 0\quad a.s.$$
  *or*
(ii) *if* ${\sigma }_{k}^{2}(l) < \beta (l)\,{\alpha }_{k}(l)$, *where* $k=1,2,\ldots ,n$, $\,\forall l\in  {\mathcal M}$. ${\bar{R}}_{0}^{\ast }:=\frac{{\sum }_{k=1}^{n}\,{\sum }_{l=1}^{L}\,{\pi }_{l}\beta (l)\,{\alpha }_{k}(l)}{{\sum }_{l=1}^{L}\,{\pi }_{l}(\mu (l)+\gamma (l)+{\sum }_{k=1}^{n}\,\frac{{\sigma }_{k}^{2}(l)}{2})} < 1$, *then the disease I*(*t*) *will die out exponentially with probability one*, *i*.*e*.,

$$\mathop{\mathrm{lim}\,{\rm{\sup }}}\limits_{t\to \infty }\,\frac{\mathrm{ln}\,I(t)}{t}\le \sum _{l=1}^{L}\,{\pi }_{l}(\mu (l)+\gamma (l)+\sum _{k=1}^{n}\,\frac{{\sigma }_{k}^{2}(l)}{2})\,({\bar{R}}_{0}^{\ast }-\mathrm{1)} < 0\quad a.s.$$

### Proof

Making use of the It $\hat{o}$’s formula to ln *I*(*t*), one has

4.1 $$\begin{array}{rcl}d\,\mathrm{ln}\,I & = & \tfrac{1}{I}\,[\frac{\beta (\xi (t))\,I\,{\sum }_{k=1}^{n}{\alpha }_{k}(\xi (t))\,{S}_{k}}{N}-(\mu (\xi (t))+\gamma (\xi (t)))I]\,dt\\  &  & -\tfrac{1}{{I}^{2}}\,\sum _{k=1}^{n}\frac{{\sigma }_{k}^{2}(\xi (t))}{2}\frac{{S}_{k}^{2}{I}^{2}}{{N}^{2}}dt+\sum _{k=1}^{n}{\alpha }_{k}(\xi (t))\frac{{S}_{k}}{N}d{B}_{k}(t)\\  & = & [\tfrac{\beta (\xi (t))\,{\sum }_{k=1}^{n}{\alpha }_{k}(\xi (t))\,{S}_{k}}{N}-\sum _{k=1}^{n}\tfrac{{\sigma }_{k}^{2}(\xi (t))}{2}\tfrac{{S}_{k}^{2}}{{N}^{2}}-(\mu (\xi (t))+\gamma (\xi (t)))]\,dt\\  &  & +\sum _{k=1}^{n}{\sigma }_{k}(\xi (t))\,\frac{{S}_{k}}{N}d{B}_{k}(t)\\  & \le  & \sum _{k=1}^{n}(\beta (\xi (t)){\alpha }_{k}(\xi (t))\frac{{S}_{k}}{N}-\frac{{\sigma }_{k}^{2}(\xi (t))}{2}\frac{{S}_{k}^{2}}{{N}^{2}})\,dt\\  &  & -(\mu (\xi (t))+\gamma (\xi (t)))\,dt\\  &  & +\sum _{k=1}^{n}{\mathop{\sigma }\limits^{\breve{}}}_{k}\frac{{S}_{k}}{N}d{B}_{k}(t).\end{array}$$

Let $\frac{{S}_{k}}{N}={z}_{k}$, *k* = 1, 2, …, *n*, and 0 < *z*_*k*_ ≤ 1, $l\in  {\mathcal M}$ we can obtain

$$\begin{array}{lll}f({z}_{k}) & := & (\beta (l){\alpha }_{k}(l){z}_{k}-\frac{{\sigma }_{k}^{2}(l)}{2}{z}_{k}^{2})\\  & = & -{(\frac{{\sigma }_{k}(l)}{\sqrt{2}}{z}_{k}-\frac{\sqrt{2}\beta (l){\alpha }_{k}(l)}{2{\sigma }_{k}(l)})}^{2}+\frac{{\beta }^{2}(l){\alpha }_{k}^{2}(l)}{2{\sigma }_{k}^{2}(l)}\\  & = & -\frac{{\sigma }_{k}^{2}(l)}{2}{({z}_{k}-\frac{\beta (l){\alpha }_{k}(l)}{{\sigma }_{k}^{2}(l)})}^{2}+\frac{{\beta }^{2}(l){\alpha }_{k}^{2}(l)}{2{\sigma }_{k}^{2}(l)}.\end{array}$$

Case 1: We can obtain:

4.2 $$f({z}_{k})\le \frac{{\beta }^{2}{(l)}_{k}^{2}(l)}{2{\alpha }_{k}^{2}(l)},$$

where *k* = 1, 2, …, *n*.

4.3 $$\begin{array}{rcl}d\,\mathrm{ln}\,I & \le  & \sum _{k=1}^{n}\frac{{\beta }^{2}(\xi (t)){\alpha }_{k}^{2}(\xi (t))}{2{\sigma }_{k}^{2}(\xi (t))}dt-(\mu (\xi (t))+\gamma (\xi (t)))\,dt\\  &  & +\sum _{k=1}^{n}{\mathop{\sigma }\limits^{\breve{}}}_{k}\frac{{S}_{k}}{N}d{B}_{k}(t)\\  & := & {\bar{R}}_{0}(\xi (t))dt+\sum _{k=1}^{n}{\mathop{\sigma }\limits^{\breve{}}}_{k}\frac{{S}_{k}}{N}d{B}_{k}(t)\end{array}$$

As ergodic properties of *ξ*(*t*), we can obtain:

$$\mathop{\mathrm{lim}\,{\rm{\sup }}}\limits_{t\to +\infty }\,\frac{1}{t}\,{\int }_{0}^{t}\,{\bar{R}}_{0}(\xi (t))dt=\sum _{l=1}^{L}\,{\pi }_{l}{\bar{R}}_{0}(l)$$

Integrating (4.3) from 0 to *t*, we have

4.4 $$\mathrm{ln}\,I(t)-\,\mathrm{ln}\,I(0)\le {\int }_{0}^{t}{\bar{R}}_{0}(\xi (t))dt+\sum _{k=1}^{n}{\mathop{\sigma }\limits^{\breve{}}}_{k}\,{\int }_{0}^{t}\frac{{S}_{k}}{N}d{B}_{k}(t).$$

An application of the strong law of large numbers (in ref.^18^) we can obtain

4.5 $$\mathop{\mathrm{lim}}\limits_{t\to \infty }\,\frac{1}{t}\,{\int }_{0}^{t}{\sigma }_{k}(\xi (r))\frac{{S}_{k}}{N}d{B}_{k}(t)=0,\quad 1\le k\le n\quad a.s\mathrm{.}.$$

Taking the superior limit on both side of (4.4) and combining with (4.5), one arrives at

$$\mathop{\mathrm{lim}\,{\rm{\sup }}}\limits_{t\to \infty }\,\frac{\mathrm{ln}\,I(t)}{t}\le \sum _{l=1}^{L}\,{\pi }_{l}(\mu (l)+\gamma (l))\,({\bar{R}}_{0}^{\ast }-1) < 0\quad a.s\mathrm{.,}$$

Case 2: When $1 < \frac{\beta (l)\,{\alpha }_{k}(l)}{{\sigma }_{k}^{2}(l)}$, that is ${\sigma }_{k}^{2}(l) < \beta (l)\,{\alpha }_{k}(l)$, then *f*(*z*_*k*_) ≤ *f*(1), we obtain:

4.6 $$f({z}_{k})\le \beta (l){\alpha }_{k}(l)-\frac{{\sigma }_{k}^{2}(l)}{2},$$

where $$\forall k=1,2,\ldots ,n$$, $$\forall l\in  {\mathcal M} $$.

4.7 $$\begin{array}{rcl}d\,\mathrm{ln}\,d & \le  & \sum _{k=1}^{n}\beta (\xi (t)){\alpha }_{k}(\xi (t))\,dt-(\mu (\xi (t))+\gamma (\xi (t))+\sum _{k=1}^{n}\frac{{\sigma }_{k}^{2}(\xi (t))}{2})\,dt\\  &  & +\sum _{k=1}^{n}{\sigma }_{k}(\xi (t))\frac{{S}_{k}}{N}d{B}_{k}(t)\\  & := & {\bar{R}}_{1}(\xi (t))\,dt+\sum _{k=1}^{n}{\sigma }_{k}(\xi (t))\frac{{S}_{k}}{N}d{B}_{k}(t)\end{array}$$

As ergodic properties of *ξ*(*t*), we can obtain:

$$\mathop{\mathrm{lim}\,{\rm{\sup }}}\limits_{t\to +\infty }\,\frac{1}{t}\,{\int }_{0}^{t}\,{\bar{R}}_{1}(\xi (t))\,dt=\sum _{l=1}^{L}\,{\pi }_{l}{\bar{R}}_{1}(l)$$

Integrating (4.7) from 0 to *t*, we have

4.8 $$\mathrm{ln}\,I(t)-\,\mathrm{ln}\,I(0)\le {\int }_{0}^{t}{\bar{R}}_{0}^{\ast }(\xi (t))\,dr+\sum _{k=1}^{n}{\int }_{0}^{t}{\sigma }_{k}(\xi (r))\frac{{S}_{k}}{N}d{B}_{k}(r).$$

An application of the strong law of large numbers (in ref.^18^) we can obtain Taking the superior limit on both side of (4.4) and combining with (4.5), one arrives at

$$\mathop{\mathrm{lim}\,{\rm{\sup }}}\limits_{t\to \infty }\,\frac{\mathrm{ln}\,I(t)}{t}\le \sum _{l=1}^{L}\,{\pi }_{l}(\mu (l)+\gamma (l)+\sum _{k=1}^{n}\,\frac{{\sigma }_{k}^{2}(l)}{2})\,({\bar{R}}_{0}^{\ast }-\mathrm{1)} < 0\quad a.s.,$$

which implies that ${\mathrm{lim}}_{t\to \infty }\,I(t)=0$ *a*.*s*. Thus the disease *I*(*t*) will tend to zero exponentially with probability one.

By system (1.8) and (1.2), it is easy to see that when ${\mathrm{lim}}_{t\to \infty }\,I(t)=0$ *a*.*s*., then ${\mathrm{lim}}_{t\to \infty }\,A(t)=0$ *a*.*s*. This completes the proof.

**Remark 4.1** Sufficient criteria of extinction are established for the HIV infectious disease in the stochastic system. Condition (1) of Theorem 4.1 tells us if the noise is strong, then the disease will die out. Condition (2) of Theorem 4.1 is to say if the noise is weak, then the disease also will die out under specific condition.

## Persistence

**Definition 5.1** *System* (1.8) *is said to be persistet in the mean if*

$$\mathop{\mathrm{lim}\,{\rm{\inf }}}\limits_{t\to \infty }\,\frac{1}{t}\,{\int }_{0}^{t}\,\frac{I(r)}{N(r)}dr > 0\quad a.s.$$

We define a parameter

5.1 $${R}_{0}^{s}:=\sum _{k=1}^{n}\tfrac{{[{\sum }_{l=1}^{L}{\pi }_{l}{({\mu }^{2}(l)\beta (l){\alpha }_{k}(l){S}_{k}^{0}(l))}^{\tfrac{1}{3}}]}^{3}}{{\sum }_{l=1}^{L}{\pi }_{l}(\mu (l)+\tfrac{{\sigma }_{k}^{2}(l)}{2})\,{\sum }_{l=1}^{L}{\pi }_{l}(\mu (l)+\gamma (l)+{\sum }_{k=1}^{n}\tfrac{{\sigma }_{k}^{2}(l)}{2})\,{\sum }_{l=1}^{L}{\pi }_{l}(\mu (l)\,{\sum }_{k=1}^{n}{S}_{k}^{0}(l))}.$$

### Theorem 5.1

*Assume that* ${R}_{0}^{s} > 1$, *then for any initial value* (*S*_1_(0), *S*_2_(0), …, *S*_*n*_(0), *I*(0), *ξ*(0)) ∈ Γ^*^ *the solution* (*S*_1_(*t*), *S*_2_(*t*), …, *S*_*n*_(*t*), *I*(*t*), *ξ*(*t*)) *of system* (*1*.*8*) *has the following property*:

5.2 $$\mathop{\mathrm{lim}\,{\rm{\inf }}}\limits_{t\to \infty }\,\frac{1}{t}\,{\int }_{0}^{t}\frac{I(r)}{N(r)}dr\ge \tfrac{{\sum }_{l=1}^{L}{\pi }_{l}(\mu (l)+\gamma (l)+{\sum }_{k=1}^{n}\tfrac{{\sigma }_{k}^{2}(l)}{2})\,({R}_{0}^{s}-1)}{\mathop{\beta }\limits^{\breve{}}\tfrac{{[{\sum }_{l=1}^{L}{\pi }_{l}{({\mu }^{2}(l)\beta (l){\alpha }_{k}(l){S}_{k}^{0}(l))}^{\tfrac{1}{3}}]}^{3}}{{[{\sum }_{l=1}^{L}{\pi }_{l}(\mu (l)+\tfrac{{\sigma }_{k}^{2}(l)}{2})]}^{2}\,{\sum }_{l=1}^{L}{\pi }_{l}(\mu (l){\sum }_{k=1}^{n}{S}_{k}^{0}(l))}{\mathop{\alpha }\limits^{\breve{}}}_{k}} > 0,$$

*where k* = 1, 2, …, *n*.

*Proof*.

$$\begin{array}{rcl} {\mathcal L} (\sum _{k=1}^{n}\,{S}_{k}+I) & = & \mu (l)\sum _{k=1}^{n}\,({S}_{k}^{0}(l)-{S}_{k})-(\mu (l)+\gamma (l))I=\mu (l)\,\sum _{k=1}^{n}\,{S}_{k}^{0}(l)\\  &  & -\mu (l)\,(\sum _{k\mathrm{=1}}^{n}{S}_{k}+I)-\gamma (l)I\\  & = & \mu (l)\,\sum _{k\mathrm{=1}}^{n}\,{S}_{k}^{0}(l)-\mu (l)N-\gamma (l)I\\ \quad \,\, {\mathcal L} (-\mathrm{ln}\,{S}_{k}) & = & -\frac{\mu (l){S}_{k}^{0}(l)}{{S}_{k}}+\mu (l)+\frac{\beta (l){\alpha }_{k}(l)I}{N}+\frac{{\sigma }_{k}^{2}(l)}{2}\frac{{I}^{2}}{{N}^{2}},\end{array}$$

where *k* = 1, 2, …, *n*,

$$ {\mathcal L} (-\mathrm{ln}\,I)=-\frac{\beta (l)\sum _{k=1}^{n}{\alpha }_{k}(l){S}_{k}}{N}+(\mu (l)+\gamma (l))+\sum _{k\mathrm{=1}}^{n}\,\frac{{\sigma }_{k}^{2}(l)}{2}\frac{{S}_{k}^{2}}{{N}^{2}}.$$

Hence we define

$$U({S}_{1},{S}_{2},\ldots ,{S}_{n},I)=-\mathrm{ln}\,I-\sum _{k=1}^{n}\,{c}_{k}\,\mathrm{ln}\,{S}_{k}+\sum _{k=1}^{n}\,{a}_{k}(\sum _{k=1}^{n}\,{S}_{k}+I),$$

with

$$\begin{array}{l}{c}_{k}=\frac{{[{\sum }_{l=1}^{L}{\pi }_{l}{({\mu }^{2}(l)\beta (l){\alpha }_{k}(l){S}_{k}^{0}(l))}^{\frac{1}{3}}]}^{3}}{{[{\sum }_{l=1}^{L}{\pi }_{l}(\mu (l)+\frac{{\sigma }_{k}^{2}(l)}{2})]}^{2}{\sum }_{l=1}^{L}{\pi }_{l}(\mu (l){\sum }_{k=1}^{n}{S}_{k}^{0}(l))},\\ {a}_{k}=\frac{{[{\sum }_{l=1}^{L}{\pi }_{l}{({\mu }^{2}(l)\beta (l){\alpha }_{k}(l){S}_{k}^{0}(l))}^{\frac{1}{3}}]}^{3}}{{\sum }_{l=1}^{L}{\pi }_{l}(\mu (l)+\frac{{\sigma }_{k}^{2}(l)}{2})\,{[{\sum }_{l=1}^{L}{\pi }_{l}(\mu (l){\sum }_{k=1}^{n}{S}_{k}^{0}(l))]}^{2}},\end{array}$$

in which *k* = 1, 2, …, *n*.

Using It $\hat{o}$’s formula and Basic inequality $\frac{a+b+c}{3}\ge \sqrt[3]{abc}$ one can write

53 $$\begin{array}{rcl} {\mathcal L} U & = & -\sum _{k\mathrm{=1}}^{n}\tfrac{\beta (l){\alpha }_{k}(l){S}_{k}}{N}+(\mu (l)+\gamma (l))-\sum _{k\mathrm{=1}}^{n}\tfrac{{c}_{k}\mu (l){S}_{k}^{0}(l)}{{S}_{k}}-\sum _{k\mathrm{=1}}^{n}{a}_{k}\mu (l)N\\  &  & +\tfrac{\beta (l)I{\sum }_{k=1}^{n}{c}_{k}{\alpha }_{k}(l)}{N}+\sum _{k\mathrm{=1}}^{n}{c}_{k}(\mu (l)+\frac{{\sigma }_{k}^{2}(l)}{2}\frac{{I}^{2}}{{N}^{2}})+\mu (l)\sum _{k\mathrm{=1}}^{n}\,{a}_{k}(\sum _{k\mathrm{=1}}^{n}{S}_{k}^{0}(l))\\  &  & -\sum _{k\mathrm{=1}}^{n}{a}_{k}\gamma (l)I+\frac{\beta (l){\alpha }_{k}(l)I}{N}+\sum _{k\mathrm{=1}}^{n}\frac{{\sigma }_{k}^{2}(l)}{2}\frac{{S}_{k}^{2}}{{N}^{2}}\\  & \le  & -\sum _{k\mathrm{=1}}^{n}\frac{\beta (l){\alpha }_{k}(l){S}_{k}}{N}+(\mu (l)+\gamma (l)+\sum _{k\mathrm{=1}}^{n}\frac{{\sigma }_{k}^{2}(l)}{2})-\sum _{k\mathrm{=1}}^{n}\frac{{c}_{k}\mu (l){S}_{k}^{0}(l)}{{S}_{k}}\\  &  & -\sum _{k\mathrm{=1}}^{n}{a}_{k}\mu (l)N+\frac{\beta (l)I{\sum }_{k=1}^{n}{c}_{k}{\alpha }_{k}(l)}{N}+\sum _{k\mathrm{=1}}^{n}{c}_{k}(\mu (l)+\frac{{\sigma }_{k}^{2}(l)}{2})\\  &  & +\mu (l)\sum _{k\mathrm{=1}}^{n}{a}_{k}(\sum _{k\mathrm{=1}}^{n}{S}_{k}^{0}(l))-\sum _{k\mathrm{=1}}^{n}{a}_{k}\gamma (l)I\\  & = & \sum _{k\mathrm{=1}}^{n}[-\tfrac{\beta (l){\alpha }_{k}(l){S}_{k}}{N}-\tfrac{{c}_{k}\mu (l){S}_{k}^{0}(l)}{{S}_{k}}-{a}_{k}\mu (l)N]+(\mu (l)+\gamma (l)+\sum _{k\mathrm{=1}}^{n}\tfrac{{\sigma }_{k}^{2}(l)}{2})\\  &  & +\tfrac{\beta (l)I{\sum }_{k=1}^{n}{c}_{k}{\alpha }_{k}(l)}{N}+\sum _{k\mathrm{=1}}^{n}{c}_{k}(\mu (l)+\frac{{\sigma }_{k}^{2}(l)}{2})+\mu (l)\sum _{k\mathrm{=1}}^{n}{a}_{k}(\sum _{k\mathrm{=1}}^{n}{S}_{k}^{0}(l))-\sum _{k\mathrm{=1}}^{n}{a}_{k}\gamma (l)I\\  & \le  & -3\sum _{k\mathrm{=1}}^{n}{({c}_{k}\beta (l){\mu }^{2}(l){\alpha }_{k}(l){S}_{k}^{0}(l){a}_{k})}^{\frac{1}{3}}+\sum _{k\mathrm{=1}}^{n}{c}_{k}(\mu (l)+\frac{{\sigma }_{k}^{2}(l)}{2})+\mu (l)\sum _{k\mathrm{=1}}^{n}{a}_{k}(\sum _{k\mathrm{=1}}^{n}{S}_{k}^{0}(l))\\  &  & +(\mu (l)+\gamma (l)+\sum _{k\mathrm{=1}}^{n}\frac{{\sigma }_{k}^{2}(l)}{2})+\frac{\beta (l)\sum _{k\mathrm{=1}}^{n}{c}_{k}{\alpha }_{k}(l)}{N}I\\  & := & {\bar{R}}_{0}(l)+\frac{\beta (l)\sum _{k\mathrm{=1}}^{n}{c}_{k}{\alpha }_{k}(l)}{N}I.\end{array}$$

By *c*_*k*_, *a*_*k*_, *k* = 1, 2, …, *n*, we obtain

$$\begin{array}{rcl}{c}_{k}\sum _{l\mathrm{=1}}^{L}{\pi }_{l}(\mu (l)+\frac{{\sigma }_{k}^{2}(l)}{2}) & = & {a}_{k}\sum _{l\mathrm{=1}}^{L}{\pi }_{l}(\mu (l)\sum _{k\mathrm{=1}}^{n}{S}_{k}^{0}(l))\\  & = & \frac{{[{\sum }_{l=1}^{L}{\pi }_{l}{({\mu }^{2}(l)\beta (l){\alpha }_{k}(l){S}_{k}^{0}(l))}^{\frac{1}{3}}]}^{3}}{{\sum }_{l=1}^{L}{\pi }_{l}(\mu (l)+\frac{{\sigma }_{k}^{2}(l)}{2}){\sum }_{l=1}^{L}{\pi }_{l}(\mu (l)\sum _{k\mathrm{=1}}^{n}{S}_{k}^{0}(l))},\end{array}$$

in which *k* = 1, 2, …, *n*.

As ergodic properties of *ξ*(*t*), we can obtain:

$$\mathop{\mathrm{lim}\,{\rm{\sup }}}\limits_{t\to +\infty }\frac{1}{t}{\int }_{0}^{t}\,{\bar{R}}_{0}(\xi (r))dr=\sum _{l\mathrm{=1}}^{L}{\pi }_{l}{\bar{R}}_{0}(l)$$

Integrating (5.3) from 0 to *t* and dividing by *t*, we can get

54 $$\begin{array}{rcl}\frac{U(t)-U\mathrm{(0)}}{t} & \le  & \frac{1}{t}{\int }_{0}^{t}{\bar{R}}_{0}(\xi (r))dr+\mathop{\beta }\limits^{\breve{}}\sum _{k\mathrm{=1}}^{n}\,{c}_{k}{\mathop{\alpha }\limits^{\breve{}}}_{k}\frac{1}{t}{\int }_{0}^{t}\,\frac{I(r)}{N(r)}dr\\  &  & +\sum _{k\mathrm{=1}}^{n}\,\frac{1}{t}{\int }_{0}^{t}\,\,{\sigma }_{k}(\xi (r))\frac{I(r)}{N(r)}d{B}_{k}(r)\\  &  & -\sum _{k\mathrm{=1}}^{n}\frac{1}{t}{\int }_{0}^{t}\,{\sigma }_{k}(\xi (r))\frac{{S}_{k}(r)}{N(r)}d{B}_{k}(r\mathrm{)}.\end{array}$$

Since ${\sum }_{k=1}^{n}\,{S}_{k}(t)+I(t)\le C$, we can obtain

55 $$\begin{array}{rcl}U(t) & = & -\mathrm{ln}\,I(t)-\sum _{k\mathrm{=1}}^{n}\,{c}_{k}\,\mathrm{ln}\,{S}_{k}(t)+\sum _{k\mathrm{=1}}^{n}{a}_{k}(\sum _{k\mathrm{=1}}^{n}{S}_{k}(t)+I(t))\\  & \ge  & -\mathrm{ln}\,I(t)-\sum _{k\mathrm{=1}}^{n}\,{c}_{k}\,\mathrm{ln}\,{S}_{k}(t)\\  & \ge  & -\mathrm{ln}\,C-\sum _{k\mathrm{=1}}^{n}\,{c}_{k}\,\mathrm{ln}\,C:=\bar{M}.\end{array}$$

An application of the strong law of large numbers (in ref.^18^) we can obtain

5.6 $$\mathop{\mathrm{lim}}\limits_{t\to \infty }\frac{1}{t}{\int }_{0}^{t}\,{\sigma }_{k}(\xi (r))\frac{{S}_{i}(r)}{N(r)}d{B}_{i}(r)=0\quad 1\le i\le n\quad a.s.$$

5.7 $$\mathop{\mathrm{lim}}\limits_{t\to \infty }\frac{1}{t}{\int }_{0}^{t}\,{\sigma }_{k}(\xi (r))\frac{I(r)}{N(r)}d{B}_{i}(r)=0$$

Taking the superior limit on both side of (5.4) and combining with (5.5), (5.6) and (5.7) one arrives at

$$\begin{array}{l}\mathop{\mathrm{lim}\,{\rm{\inf }}}\limits_{t\to \infty }\frac{1}{t}{\int }_{0}^{t}\frac{I(r)}{N(r)}dr\\ \begin{array}{ll}\quad \ge  & -\frac{{\sum }_{l=1}^{L}{\pi }_{l}{\bar{R}}_{0}(l)}{\mathop{\beta }\limits^{\breve{}}{\sum }_{k=1}^{n}{c}_{k}{\mathop{\alpha }\limits^{\breve{}}}_{k}}\\ \quad \ge  & \frac{1}{\mathop{\beta }\limits^{\breve{}}{\sum }_{k=1}^{n}{c}_{k}{\mathop{\alpha }\limits^{\breve{}}}_{k}}\{\sum _{k\mathrm{=1}}^{n}\frac{3{[{\sum }_{l=1}^{L}{\pi }_{l}{({\mu }^{2}(l)\beta (l){\alpha }_{k}(l){S}_{k}^{0}(l))}^{\frac{1}{3}}]}^{3}}{{\sum }_{l=1}^{L}{\pi }_{l}(\mu (l)+\frac{{\sigma }_{k}^{2}(l)}{2})\,{\sum }_{l=1}^{L}{\pi }_{l}(\mu (l){\sum }_{k=1}^{n}{S}_{k}^{0}(l))}\\  & -\sum _{k\mathrm{=1}}^{n}\tfrac{2{[{\sum }_{l=1}^{L}{\pi }_{l}{({\mu }^{2}(l)\beta (l){\alpha }_{k}(l){S}_{k}^{0}(l))}^{\tfrac{1}{3}}]}^{3}}{{\sum }_{l=1}^{L}{\pi }_{l}(\mu (l)+\tfrac{{\sigma }_{k}^{2}(l)}{2})\,{\sum }_{l=1}^{L}{\pi }_{l}(\mu (l){\sum }_{k=1}^{n}{S}_{k}^{0}(l))}-\sum _{l\mathrm{=1}}^{L}\,{\pi }_{l}(\mu (l)+\gamma (l)\\  & +\sum _{k\mathrm{=1}}^{n}\frac{{\sigma }_{k}^{2}(l)}{2})\}\\ \quad \ge  & \frac{1}{\mathop{\beta }\limits^{\breve{}}\,{\sum }_{k=1}^{n}{c}_{k}{\mathop{\alpha }\limits^{\breve{}}}_{k}}\{\sum _{k\mathrm{=1}}^{n}\tfrac{{[{\sum }_{l=1}^{L}{\pi }_{l}{({\mu }^{2}(l)\beta (l){\alpha }_{k}(l){S}_{k}^{0}(l))}^{\tfrac{1}{3}}]}^{3}}{{\sum }_{l=1}^{L}{\pi }_{l}(\mu (l)+\tfrac{{\sigma }_{k}^{2}(l)}{2})\,{\sum }_{l=1}^{L}{\pi }_{l}(\mu (l){\sum }_{k=1}^{n}{S}_{k}^{0}(l))}-\sum _{l\mathrm{=1}}^{L}{\pi }_{l}(\mu (l)+\gamma (l)\\  & +\sum _{k\mathrm{=1}}^{n}\frac{{\sigma }_{k}^{2}(l)}{2})\}\\ \quad \ge  & \frac{{\sum }_{l=1}^{L}{\pi }_{l}(\mu (l)+\gamma (l)+{\sum }_{k=1}^{n}\frac{{\sigma }_{k}^{2}(l)}{2})\,({R}_{0}^{s}-\mathrm{1)}}{\mathop{\beta }\limits^{\breve{}}{\sum }_{k=1}^{n}{c}_{k}{\mathop{\alpha }\limits^{\breve{}}}_{k}}.\end{array}\end{array}$$

Therefore, by the condition ${R}_{0}^{s} > 1$, we have assertion (5.2). This complete the proof of Theorem 5.1.

**Remark 5.1** Theorem 5.1 tells us if the the condition of Theorem 5.1 is satisfied, then the disease will proceed. The conditions for the persistence of HIV infectious disease in the stochastic system are sufficient but not necessary.

## Positive Recurrence

In this section, we show the persistence of the disease in the population, but from another point of view. Precisely, we find a domain $D\subset {{\rm{\Gamma }}}^{\ast }$ in which the process (*S*_1_(*t*), *S*_2_(*t*), …, *S*_*n*_(*t*), *I*(*t*)) is positive recurrent. Generally, the process ${X}_{t}^{x}$ where *X*_0_ = *x* is recurrent with respect to *D*, if for any $x\notin D$, ${\mathbb{P}}({\tau }_{D} < +\infty )=1$, where *τ*_*D*_ is the hitting time of *D* for the process ${X}_{t}^{x}$, that is

$${\tau }_{D}={\rm{\inf }}\,\{t > \mathrm{0,}\,{X}_{t}^{x}\in D\}.$$

The process ${X}_{t}^{x}$ is said to be positive recurrent with respect to *D* if ${\mathbb{E}}({\tau }_{D}) < +\infty$, for any $x\notin D$.

### Theorem 6.1

*Let* (*S*_1_(*t*), *S*_2_(*t*), …, *S*_*n*_(*t*), *I*(*t*), *ξ*(*t*)) *be the solution of system* (*1*.*8*) *with initial value* $({S}_{1}\mathrm{(0)},{S}_{2}\mathrm{(0)},\ldots ,$ ${S}_{n}\mathrm{(0)},I\mathrm{(0)},\xi \mathrm{(0))}\in {{\rm{\Gamma }}}^{\ast }\times  {\mathcal M}$. *Assume that* ${R}_{0}^{s} > 1$ (*defined by Section 4*), *then* (*S*_1_(*t*), *S*_2_(*t*), …, *S*_*n*_(*t*), *I*(*t*), *ξ*(*t*)) *is positive recurrent with respect to the domain* $D\times  {\mathcal M}$, *where*

$$\begin{array}{rcl}D & = & \{({S}_{1},{S}_{2},\ldots ,{S}_{n},I)\in {{\rm{\Gamma }}}^{\ast },n{\varepsilon }_{1} < \sum _{k\mathrm{=1}}^{n}{S}_{k} < \sum _{k\mathrm{=1}}^{n}{\mathop{S}\limits^{\breve{}}}_{k}^{0}-{\varepsilon }_{2}-{\varepsilon }_{3},{\varepsilon }_{2} < I < \sum _{k\mathrm{=1}}^{n}{\mathop{S}\limits^{\breve{}}}_{k}^{0}\\  &  & -n{\varepsilon }_{1}-{\varepsilon }_{3},n{\varepsilon }_{1}+{\varepsilon }_{2} < \sum _{k=1}^{n}{S}_{k}+I < \sum _{k\mathrm{=1}}^{n}{\mathop{S}\limits^{\breve{}}}_{k}^{0}-{\varepsilon }_{3}\},\end{array}$$

*in which ε*_1_,*ε*_2_,*ε*_3_ *are sufficiently small constants*.

*Proof*. Since the coefficients of (1.8) are constants, it is not difficult to show that they satisfy (5.1), (5.2). For all initial value $({S}_{1}\mathrm{(0)},{S}_{2}\mathrm{(0)},\ldots ,{S}_{n}\mathrm{(0)},I\mathrm{(0)},\xi \mathrm{(0))}\in {{\rm{\Gamma }}}^{\ast }\times  {\mathcal M}$, the solution of (1.8) is regular by Theorem (3.1).

Defining a *C* ^2^–function

$$\widehat{V}=M(U+w(l))-\sum _{k\mathrm{=1}}^{n}\,\mathrm{ln}\,{S}_{k}-\,\mathrm{ln}(\sum _{k\mathrm{=1}}^{n}{\mathop{S}\limits^{\breve{}}}_{k}^{0}-\sum _{k\mathrm{=1}}^{n}{S}_{k}-I),$$

in which *U*(*S*_1_, *S*_2_, …, *S*_*n*_, *I*) is defined by section (5). Let (*w*(1), *w*(2), …, *w*(*L*))^*T*^ be the solution of the following Poisson system

6.1 $${\rm{\Gamma }}w=\sum _{h\mathrm{=1}}^{L}\,{\pi }_{h}{\bar{R}}_{0}(h)\,(\begin{array}{l}1\\ 1\\ \vdots \\ 1\end{array})-{\bar{R}}_{0},$$

where $${\bar{R}}_{0}={({\bar{R}}_{0}\mathrm{(1)},{\bar{R}}_{0}\mathrm{(2)},\ldots ,{\bar{R}}_{0}(L))}^{T}$$ then

$$\sum _{h\in  {\mathcal M} }\,{\gamma }_{lh}w(h)+{\bar{R}}_{0}(l)=\sum _{h\mathrm{=1}}^{L}\,{\pi }_{h}{\bar{R}}_{0}(h),\,l=1,2,\ldots ,L.$$

By *c*_*k*_, *a*_*k*_, *k* = 1, 2, …, *n*, we obtain

$$\begin{array}{rcl}{c}_{k}\sum _{l\mathrm{=1}}^{L}{\pi }_{l}(\mu (l)+\frac{{\sigma }_{k}^{2}(l)}{2}) & = & {a}_{k}\sum _{l\mathrm{=1}}^{L}{\pi }_{l}(\mu (l)\sum _{k\mathrm{=1}}^{n}{S}_{k}^{0}(l))\\  & = & \frac{{[{\sum }_{l=1}^{L}{\pi }_{l}{({\mu }^{2}(l)\beta (l){\alpha }_{k}(l){S}_{k}^{0}(l))}^{\frac{1}{3}}]}^{3}}{{\sum }_{l=1}^{L}{\pi }_{l}(\mu (l)+\frac{{\sigma }_{k}^{2}(l)}{2})\,{\sum }_{l=1}^{L}{\pi }_{l}(\mu (l){\sum }_{k=1}^{n}{S}_{k}^{0}(l))},\end{array}$$

in which *k* = 1, 2, …, *n*. Then we get

$$\begin{array}{l} {\mathcal L} (U+w(l))\\ \begin{array}{ll}\quad \le  & \sum _{l\mathrm{=1}}^{L}{\pi }_{l}{\bar{R}}_{0}(l)+\frac{\beta (l){\sum }_{k=1}^{n}{c}_{k}{\alpha }_{k}(l)}{N}I\\ \quad \le  & \sum _{k\mathrm{=1}}^{n}\tfrac{-3{[{\sum }_{l=1}^{L}{\pi }_{l}{({\mu }^{2}(l)\beta (l){\alpha }_{k}(l){S}_{k}^{0}(l))}^{\tfrac{1}{3}}]}^{3}}{{\sum }_{l=1}^{L}{\pi }_{l}(\mu (l)+\tfrac{{\sigma }_{k}^{2}(l)}{2})\,{\sum }_{l=1}^{L}{\pi }_{l}(\mu (l){\sum }_{k=1}^{n}{S}_{k}^{0}(l))}\\  & +\sum _{k\mathrm{=1}}^{n}\tfrac{2{[{\sum }_{l=1}^{L}{\pi }_{l}{({\mu }^{2}(l)\beta (l){\alpha }_{k}(l){S}_{k}^{0}(l))}^{\tfrac{1}{3}}]}^{3}}{{\sum }_{l=1}^{L}{\pi }_{l}(\mu (l)+\tfrac{{\sigma }_{k}^{2}(l)}{2})\,{\sum }_{l=1}^{L}{\pi }_{l}(\mu (l){\sum }_{k=1}^{n}{S}_{k}^{0}(l))}+\sum _{l\mathrm{=1}}^{L}{\pi }_{l}(\mu (l)+\gamma (l)+\sum _{k\mathrm{=1}}^{n}\frac{{\sigma }_{k}^{2}(l)}{2})\\  & +\frac{\mathop{\beta }\limits^{\breve{}}{\sum }_{k=1}^{n}{c}_{k}{\mathop{\alpha }\limits^{\breve{}}}_{k}}{N}I\\ \quad = & -\sum _{k\mathrm{=1}}^{n}\tfrac{{[{\sum }_{l=1}^{L}{\pi }_{l}{({\mu }^{2}(l)\beta (l){\alpha }_{k}(l){S}_{k}^{0}(l))}^{\tfrac{1}{3}}]}^{3}}{{\sum }_{l=1}^{L}{\pi }_{l}(\mu (l)+\tfrac{{\sigma }_{k}^{2}(l)}{2})\,{\sum }_{l=1}^{L}{\pi }_{l}(\mu (l){\sum }_{k=1}^{n}{S}_{k}^{0}(l))}+\sum _{l\mathrm{=1}}^{L}{\pi }_{l}(\mu (l)+\gamma (l)+\sum _{k\mathrm{=1}}^{n}\frac{{\sigma }_{k}^{2}(l)}{2})\\  & +\frac{\mathop{\beta }\limits^{\breve{}}\sum _{k\mathrm{=1}}^{n}{c}_{k}{\mathop{\alpha }\limits^{\breve{}}}_{k}}{N}I\\ \quad \le  & -\sum _{l\mathrm{=1}}^{L}{\pi }_{l}(\mu (l)+\gamma (l)+\sum _{k\mathrm{=1}}^{n}\frac{{\sigma }_{k}^{2}(l)}{2})\,({R}_{0}^{s}-\mathrm{1)}+\frac{\mathop{\beta }\limits^{\breve{}}{\sum }_{k=1}^{n}{c}_{k}{\mathop{\alpha }\limits^{\breve{}}}_{k}}{N}I\end{array}\end{array}$$

in which ${R}_{0}^{s}$ is defined in (5.1). And the following condition for *M* > 0 is satisfied

62 $$-M\lambda +\sum _{k\mathrm{=1}}^{n}\frac{{\mathop{\sigma }\limits^{\breve{}}}_{k}^{2}}{2}+(n+\mathrm{1)}\mathop{\mu }\limits^{\breve{}}=-\mathrm{2,}$$

$$\lambda =\sum _{l\mathrm{=1}}^{L}{\pi }_{l}(\mu (l)+\gamma (l)+\sum _{k\mathrm{=1}}^{n}\frac{{\sigma }_{k}^{2}(l)}{2})\,({R}_{0}^{s}-\mathrm{1)} > 0.$$

It is easy to check that

$$\mathop{\mathrm{lim}\,{\rm{\inf }}}\limits_{\begin{array}{c}{\sum }_{k=1}^{n}{S}_{k}(t)+I(t)\to \sum _{k\mathrm{=1}}^{n}{\sum }_{k=1}^{n}{\mathop{S}\limits^{\breve{}}}_{k}^{0}\\ t\to +\infty \end{array}}\,\widehat{V}({S}_{1},{S}_{2},\ldots ,{S}_{n},I,l)=+\infty .$$

In addition, $\widehat{V}({S}_{1},{S}_{2},\ldots ,{S}_{n},I,l)$ is a continuous function on ${\bar{U}}_{k}$. Therefore $\widehat{V}(({S}_{1},{S}_{2},\ldots ,{S}_{n},I,l)$ has a minimum value point $({\bar{S}}_{1},{\bar{S}}_{2},\ldots ,{\bar{S}}_{n},\bar{I},l)$ in the interior of ${{\rm{\Gamma }}}^{\ast }\times  {\mathcal M}$. Then we define a nonnegative *C* ^2^–function V: ${{\rm{\Gamma }}}^{\ast }\times  {\mathcal M} \to {\mathbb{R}}$ as follows

$$V({S}_{1},{S}_{2},\ldots ,{S}_{n},I,l)=\widehat{V}({S}_{1},{S}_{2},\ldots ,{S}_{n},I,l)-\widehat{V}({\bar{S}}_{1},{\bar{S}}_{2},\ldots ,{\bar{S}}_{n},\bar{I},l\mathrm{)}.$$

The differential operator ${\mathcal L}$ acting on the function *V* leads to

$$\begin{array}{rcl} {\mathcal L} V & \le  & M[-\sum _{l\mathrm{=1}}^{L}{\pi }_{l}(\mu (l)+\gamma (l)+\sum _{k\mathrm{=1}}^{n}\frac{{\sigma }_{k}^{2}(l)}{2})\,({R}_{0}^{s}-\mathrm{1)}+\frac{\mathop{\beta }\limits^{\breve{}}{\sum }_{k=1}^{n}{c}_{k}{\mathop{\alpha }\limits^{\breve{}}}_{k}}{L}I]\\  &  & +\frac{\mathop{\beta }\limits^{\breve{}}{\sum }_{k=1}^{n}{\mathop{\alpha }\limits^{\breve{}}}_{k}}{N}I-\sum _{k\mathrm{=1}}^{n}\frac{\hat{\mu }{\hat{S}}_{k}^{0}}{{S}_{k}}+\sum _{k\mathrm{=1}}^{n}\frac{{\mathop{\sigma }\limits^{\breve{}}}_{k}^{2}}{2}\frac{{I}^{2}}{{N}^{2}}\\  &  & +(n+\mathrm{1)}\mathop{\mu }\limits^{\breve{}}-\frac{\hat{\gamma }I}{{\sum }_{k=1}^{n}{\mathop{S}\limits^{\breve{}}}_{k}^{0}-N}\\  & \le  & M[-\sum _{l\mathrm{=1}}^{L}{\pi }_{l}(\mu (l)+\gamma (l)+\sum _{k\mathrm{=1}}^{n}\frac{{\sigma }_{k}^{2}(l)}{2})\,({R}_{0}^{s}-\mathrm{1)}+\frac{\mathop{\beta }\limits^{\breve{}}{\sum }_{k=1}^{n}{c}_{k}{\mathop{\alpha }\limits^{\breve{}}}_{k}}{N}I]\\  &  & +\frac{\mathop{\beta }\limits^{\breve{}}{\sum }_{k=1}^{n}{\mathop{\alpha }\limits^{\breve{}}}_{k}}{N}I-\sum _{k\mathrm{=1}}^{n}\frac{\hat{\mu }{\hat{S}}_{k}^{0}}{{S}_{k}}+\sum _{k\mathrm{=1}}^{n}\frac{{\mathop{\sigma }\limits^{\breve{}}}_{k}^{2}}{2}\\  &  & +(n+\mathrm{1)}\mathop{\mu }\limits^{\breve{}}-\frac{\hat{\gamma }I}{{\sum }_{k=1}^{n}{\mathop{S}\limits^{\breve{}}}_{k}^{0}-N}\\  & := & -M\lambda +\frac{\mathop{\beta }\limits^{\breve{}}(M{\sum }_{k=1}^{n}{c}_{k}{\mathop{\alpha }\limits^{\breve{}}}_{k}+{\sum }_{k=1}^{n}{\mathop{\alpha }\limits^{\breve{}}}_{k})}{{\sum }_{k=1}^{n}{S}_{k}+I}I\\  &  & -\sum _{k\mathrm{=1}}^{n}\frac{\hat{\mu }{\hat{S}}_{k}^{0}}{{S}_{k}}-\frac{\hat{\gamma }I}{{\sum }_{k=1}^{n}{\mathop{S}\limits^{\breve{}}}_{k}^{0}-N}+(n+\mathrm{1)}\mathop{\mu }\limits^{\breve{}}+\sum _{k\mathrm{=1}}^{n}\frac{{\mathop{\sigma }\limits^{\breve{}}}_{k}^{2}}{2}.\end{array}$$

Consider the bounded open subset

$$\begin{array}{rcl}D & = & \{({S}_{1},{S}_{2},\ldots ,{S}_{n},I)\in {{\mathbb{R}}}_{+}^{n+1},n{\varepsilon }_{1} < \sum _{k\mathrm{=1}}^{n}{S}_{k} < \sum _{k\mathrm{=1}}^{n}{\mathop{S}\limits^{\breve{}}}_{k}^{0}-{\varepsilon }_{2}-{\varepsilon }_{3},{\varepsilon }_{2} < I < \sum _{k\mathrm{=1}}^{n}{\mathop{S}\limits^{\breve{}}}_{k}^{0}\\  &  & -n{\varepsilon }_{1}-{\varepsilon }_{3},n{\varepsilon }_{1}+{\varepsilon }_{2} < \sum _{k\mathrm{=1}}^{n}{S}_{k}+I < \sum _{k\mathrm{=1}}^{n}{\mathop{S}\limits^{\breve{}}}_{k}^{0}-{\varepsilon }_{3}\},\end{array}$$

and *ε*_*i*_ > 0 (*i* = 1, 2, 3) are sufficiently small constants. In the set Γ^*^\*D*, we can get *ε*_*i*_(*i* = 1, 2, 3) sufficiently small such that the following conditions hold

6.3 $$-\frac{\hat{\mu }{\hat{S}}_{k}^{0}}{{\varepsilon }_{1}}+\widehat{K}\le -1\quad k=1,2,\ldots ,n,$$

6.4 $${\varepsilon }_{2}={(n{\varepsilon }_{1})}^{2}.$$

6.5 $${\varepsilon }_{3}={\varepsilon }_{2}^{2}.$$

6.6 $$\mathop{\beta }\limits^{\breve{}}(M\sum _{k\mathrm{=1}}^{n}{c}_{k}{\mathop{\alpha }\limits^{\breve{}}}_{k}+\sum _{k\mathrm{=1}}^{n}{\mathop{\alpha }\limits^{\breve{}}}_{k})n{\varepsilon }_{1}\le 1.$$

6.7 $$\widehat{K}=\mathop{\beta }\limits^{\breve{}}(M\sum _{k\mathrm{=1}}^{n}{c}_{k}{\mathop{\alpha }\limits^{\breve{}}}_{k}+\sum _{k\mathrm{=1}}^{n}{\mathop{\alpha }\limits^{\breve{}}}_{k})-2.$$

6.8 $$\widehat{K}-\frac{\hat{\gamma }}{{\varepsilon }_{2}}\le -1.$$

For the purpose of convenience, we can divide Γ^*^\*D* into the following 2*n* + 2 domains,

$${D}_{k}=\mathrm{\{0} < {S}_{k}\le {\varepsilon }_{1}\},\quad k=1,2,\ldots ,n.$$

$${D}_{n+1}=\mathrm{\{0} < I\le {\varepsilon }_{2},{\varepsilon }_{1}\le {S}_{k}\,1\le k\le n\}.$$

$${D}_{n+2}=\{{\varepsilon }_{2}\le I\le \sum _{k\mathrm{=1}}^{n}{\mathop{S}\limits^{\breve{}}}_{k}^{0}-{\varepsilon }_{3},\sum _{k\mathrm{=1}}^{n}{\mathop{S}\limits^{\breve{}}}_{k}^{0}-{\varepsilon }_{3}\le \sum _{k\mathrm{=1}}^{n}{S}_{k}+I\}.$$

Clearly, ${D}^{c}={D}_{1}\cup {D}_{2}\cup {D}_{3}\cup \cdots \cup {D}_{n+2}$. Next we will prove that ${\mathcal L} V({S}_{1},{S}_{2},\ldots ,{S}_{n},I,l)\le -1$ on ${D}^{c}\times  {\mathcal M}$, which is equivalent to show it on the above *n* + 2 domains.

Case 1. If (*S*_1_, *S*_2_, …, *S*_*n*_, *I*) ∈ *D*_*k*_, (*k* = 1, 2, …, *n*), then

6.9 $$\begin{array}{rcl} {\mathcal L} V & \le  & -M\lambda +\frac{\mathop{\beta }\limits^{\breve{}}(M{\sum }_{k=1}^{n}{c}_{k}{\mathop{\alpha }\limits^{\breve{}}}_{k}+{\sum }_{k=1}^{n}{\mathop{\alpha }\limits^{\breve{}}}_{k})}{{\sum }_{k=1}^{n}{S}_{k}+I}I-\frac{\hat{\mu }{\hat{S}}_{k}^{0}}{{S}_{k}}\\  &  & +(n+\mathrm{1)}\mathop{\mu }\limits^{\breve{}}+\sum _{k\mathrm{=1}}^{n}\frac{{\mathop{\sigma }\limits^{\breve{}}}_{k}^{2}}{2}\\  & \le  & \widehat{K}-\frac{\hat{\mu }{\hat{S}}_{k}^{0}}{{S}_{k}}\\  & \le  & \widehat{K}-\frac{\hat{\mu }{\hat{S}}_{k}^{0}}{{\varepsilon }_{1}}.\end{array}$$

In view of (6.3), one has

$$ {\mathcal L} V\le -1\,{\rm{for}}\,{\rm{any}}\,({S}_{1},{S}_{2},\ldots ,{S}_{n},I,l)\in {D}_{k}\times  {\mathcal M} ,(k=1,2,\ldots ,n\mathrm{)}.$$

Case 2. If (*S*_1_, *S*_2_, …, *S*_*n*_, *I*) ∈ *D*_*n*+1_, then

$$\begin{array}{rcl} {\mathcal L} V & \le  & -M\lambda +\frac{\mathop{\beta }\limits^{\breve{}}(M{\sum }_{k=1}^{n}{c}_{k}{\mathop{\alpha }\limits^{\breve{}}}_{k}+{\sum }_{k=1}^{n}{\mathop{\alpha }\limits^{\breve{}}}_{k})}{{\sum }_{k=1}^{n}{S}_{k}+I}I+(n+\mathrm{1)}\mathop{\mu }\limits^{\breve{}}+\sum _{k\mathrm{=1}}^{n}\frac{{\mathop{\sigma }\limits^{\breve{}}}_{k}^{2}}{2}\\  & \le  & -M\lambda +\frac{\mathop{\beta }\limits^{\breve{}}(M{\sum }_{k=1}^{n}{c}_{k}{\mathop{\alpha }\limits^{\breve{}}}_{k}+{\sum }_{k=1}^{n}{\mathop{\alpha }\limits^{\breve{}}}_{k}){\varepsilon }_{2}}{n{\varepsilon }_{1}}+(n+\mathrm{1)}\mathop{\mu }\limits^{\breve{}}+\sum _{k\mathrm{=1}}^{n}\frac{{\mathop{\sigma }\limits^{\breve{}}}_{k}^{2}}{2}.\end{array}$$

According to (6.4) one can see that

6.10 $$ {\mathcal L} V\le -M\lambda +\mathop{\beta }\limits^{\breve{}}(M\sum _{k\mathrm{=1}}^{n}{c}_{k}{\mathop{\alpha }\limits^{\breve{}}}_{k}+\sum _{k\mathrm{=1}}^{n}{\mathop{\alpha }\limits^{\breve{}}}_{k})n{\varepsilon }_{1}+(n+\mathrm{1)}\mathop{\mu }\limits^{\breve{}}+\sum _{k\mathrm{=1}}^{n}\frac{{\mathop{\sigma }\limits^{\breve{}}}_{k}^{2}}{2}.$$

Combining with (6.2) and (6.6), one has for sufficiently small *ε*_1_,

$$ {\mathcal L} V\le -1\,{\rm{for}}\,{\rm{any}}\,({S}_{1},{S}_{2},\ldots ,{S}_{n},I,l)\in {D}_{n+1}\times  {\mathcal M} .$$

Case 3. If (*S*_1_, *S*_2_, …, *S*_*n*_, *I*) ∈ *D*_*n*+2_, then

6.11 $$\begin{array}{rcl} {\mathcal L} V & \le  & -M\lambda +\frac{\mathop{\beta }\limits^{\breve{}}(M{\sum }_{k=1}^{n}{c}_{k}{\mathop{\alpha }\limits^{\breve{}}}_{k}+{\sum }_{k=1}^{n}{\mathop{\alpha }\limits^{\breve{}}}_{k})}{{\sum }_{k=1}^{n}{S}_{k}+I}I+(n+\mathrm{1)}\mathop{\mu }\limits^{\breve{}}+\sum _{k\mathrm{=1}}^{n}\frac{{\mathop{\sigma }\limits^{\breve{}}}_{k}^{2}}{2}-\frac{\hat{\gamma }I}{\sum _{k\mathrm{=1}}^{n}{S}_{k}^{0}-N}\\  & \le  & \widehat{K}-\frac{\hat{\gamma }I}{{\sum }_{k=1}^{n}{S}_{k}^{0}-N}\\  & \le  & \widehat{K}-\frac{\hat{\gamma }{\varepsilon }_{2}}{{\varepsilon }_{3}}\\  & \le  & \widehat{K}-\frac{\hat{\gamma }}{{\varepsilon }_{2}}.\end{array}$$

In view of (6.8), one has

$$ {\mathcal L} V\le -1\,{\rm{for}}\,{\rm{any}}\,({S}_{1},{S}_{2},\ldots ,{S}_{n},I,l)\in {D}_{n+2}\times  {\mathcal M} .$$

Obviously, from (6.9), (6.10), and (6.11) one can obtain that for a sufficiently small *ε*_*i*_(*i* = 1, 2, 3),

6.12 $$ {\mathcal L} V\le -1\,{\rm{for}}\,{\rm{any}}\,({S}_{1},{S}_{2},\ldots ,{S}_{n},I,l)\in {D}^{c}\times  {\mathcal M} .$$

Now, let (*S*_1_(0), *S*_2_(0), …, *S*_*n*_(0), *I*(0)) ∈ *D* ^*c*^. Thanks to the generalized It$\hat{o}$ formula established by Skorokhod^39^ (Lemma 3, Pages 104) and using (6.12), we obtain

$$\begin{array}{l}{\mathbb{E}}[V({S}_{1}({\tau }_{D}),\,{S}_{2}({\tau }_{D}),\ldots ,\,{S}_{n}({\tau }_{D}),\,I({\tau }_{D}),\,\xi ({\tau }_{D}))]-V({S}_{1}\mathrm{(0),}\,{S}_{2}\mathrm{(0),}\ldots ,\,{S}_{n}\mathrm{(0),}\,I\mathrm{(0),}\,\xi \mathrm{(0))}\\ \quad ={\mathbb{E}}{\int }_{0}^{{\tau }_{D}} {\mathcal L} V({S}_{1}(t),\,{S}_{2}(t),\ldots ,{S}_{n}(t),\,I(t),\,\xi (t))dt\\ \quad \le -{\mathbb{E}}({\tau }_{D}\mathrm{)}.\end{array}$$

Thus, by the positivity of *V*, one can deduce that

$${\mathbb{E}}({\tau }_{D})\le V({S}_{1}\mathrm{(0),}\,{S}_{2}\mathrm{(0),}\ldots ,\,{S}_{n}\mathrm{(0),}I\mathrm{(0),}\,\xi \mathrm{(0))}.$$

The proof is complete.

**Remark 6.1** Theorem 6.1 tells us if the condition of Theorem 6.1 satisfied, then system (1.8) correspond to the boundedness of the disease to corresponding deterministic system. That is to say the disease can not spread to whole susceptible population or go to extinct.

## Conclusion

In this paper, we extended the classical DS-I-A epidemic model from a deterministic framework to a stochastic one by incorporating both white and color environmental noise. we have looked at the long-term behavior of our stochastic DS-I-A epidemic model. We established conditions for extinction and persistence of disease which both are sufficient but not necessary. We also proved that the DS-I-A model (1.8) is positive recurrent. We first discussed the conditions for extinction of disease in Section 4, it means that the disease can not spread or be endemic disease. Furthermore, we discussed the condition persistence of disease and if the disease proceed, we explore long-term behavior of the disease in Section 5 and Section 6. That is to say the disease is bounded, namely the disease will be endemic and can not spread to whole susceptible population or go to extinct.

It is interesting to find that if condition of Theorem 5.1 is satisfied, then the equation in (1.8) which *k* = 1, 2, is stochastically permanent, respectively. Hence Theorem 5.1 tells us that if every individual equation is stochastically permanent, then as the result of Markovian switching, the overall behavior, i.e. SDE (1.8), remains stochastically permanent. On the other hand, if condition of Theorem 4.1 is satisfied, for some $l\in  {\mathcal M}$, then every individual in (1.8) is extinctive. Hence Theorem 4.1 tells us that if every individual is extinctive, then as the result of Markovian switching, the overall behavior of SDE (1.8) remains extinctive. However, Theorems 4.1 and 5.1 provide a more interesting result that if some individual equations in (1.8) are stochastically permanent while some are extinctive, again as the result of Markovian switching, the overall behavior of SDE (1.8) may be stochastically permanent or extinctive, depending on the sign of the stationary distribution (*π*_1_,…,*π*_*L*_) of the Markov chain *r*(*t*).

Thus the results show that the stationary distribution (*π*_1_,…,*π*_*L*_) of the Markov chain *r*(*t*) plays a very important role in determining extinction or persistence of the epidemic in the population. We derived explicit conditions of extinction or persistence of the epidemic:

1. If following conditions satisfied:

(i) ${\bar{R}}_{0}^{\ast }:=\frac{{\sum }_{k=1}^{n}\,{\sum }_{l=1}^{L}\,{\pi }_{l}\frac{{\beta }^{2}(l)\,{\alpha }_{k}^{2}(l)}{2{\sigma }_{k}^{2}(l)}}{{\sum }_{l=1}^{L}\,{\pi }_{l}(\mu (l)+\gamma (l))} < 1$, then the disease *I*(*t*) will die out exponentially with probability one, i.e.,
  $$\mathop{\mathrm{lim}\,{\rm{\sup }}}\limits_{t\to \infty }\,\frac{\mathrm{ln}\,I(t)}{t}\le \sum _{l\mathrm{=1}}^{L}{\pi }_{l}(\mu (l)+\gamma (l))\,({\hat{R}}_{0}^{s}-\mathrm{1)} < 0\quad a.s.$$
  or
(ii) if ${\sigma }_{k}^{2}(l) < \beta (l)\,{\alpha }_{k}(l)$, where $k=1,2,\ldots ,n$, $\forall l\in  {\mathcal M}$. ${\bar{R}}_{0}^{\ast }:=\frac{{\sum }_{k=1}^{n}\,{\sum }_{l=1}^{L}\,{\pi }_{l}\beta (l)\,{\alpha }_{k}(l)}{{\sum }_{l=1}^{L}\,{\pi }_{l}(\mu (l)+\gamma (l)+{\sum }_{k=1}^{n}\,\frac{{\sigma }_{k}^{2}(l)}{2})} < 1$, then the disease *I*(*t*) will die out exponentially with probability one, i.e.,

$$\mathop{\mathrm{lim}\,{\rm{\sup }}}\limits_{t\to \infty }\,\frac{\mathrm{ln}\,I(t)}{t}\le \sum _{l\mathrm{=1}}^{L}{\pi }_{l}(\mu (l)+\gamma (l)+\sum _{k\mathrm{=1}}^{n}\frac{{\sigma }_{k}^{2}(l)}{2})\,({\hat{R}}_{0}^{s}-\mathrm{1)} < 0\quad a.s.$$

That is to say the disease will die out. It means that the disease can not spread or be endemic disease.

(2) Let (*S*_1_(*t*), *S*_2_(*t*), …, *S*_*n*_(*t*), *I*(*t*), *ξ*(*t*)) be the solution of system (1.8) with initial value $({S}_{1}\mathrm{(0)},{S}_{2}\mathrm{(0)},\ldots ,{S}_{n}\mathrm{(0)},I\mathrm{(0)},\xi \mathrm{(0))}\in {{\rm{\Gamma }}}^{\ast }\times  {\mathcal M}$. Assume that ${R}_{0}^{s} > 1$, where

$${R}_{0}^{s}:=\sum _{k\mathrm{=1}}^{n}\tfrac{{[{\sum }_{l=1}^{L}{\pi }_{l}{({\mu }^{2}(l)\beta (l){\alpha }_{k}(l){S}_{k}^{0}(l))}^{\tfrac{1}{3}}]}^{3}}{{\sum }_{l=1}^{L}{\pi }_{l}(\mu (l)+\tfrac{{\sigma }_{k}^{2}(l)}{2})\,{\sum }_{l=1}^{L}{\pi }_{l}(\mu (l)+\gamma (l)+{\sum }_{k=1}^{n}\tfrac{{\sigma }_{k}^{2}(l)}{2})\,{\sum }_{l=1}^{L}{\pi }_{l}(\mu (l){\sum }_{k=1}^{n}{S}_{k}^{0}(l))},$$

then (*S*_1_(*t*), *S*_2_(*t*), …, *S*_*n*_(*t*), *I*(*t*), *ξ*(*t*)) is persistence and is positive recurrent with respect to the domain $D\times  {\mathcal M}$, where

$$\begin{array}{rcl}D & = & \{({S}_{1},{S}_{2},\ldots ,{S}_{n},I)\in {{\rm{\Gamma }}}^{\ast },n{\varepsilon }_{1} < \sum _{k\mathrm{=1}}^{n}{S}_{k} < \sum _{k\mathrm{=1}}^{n}{\mathop{S}\limits^{\breve{}}}_{k}^{0}-{\varepsilon }_{2}-{\varepsilon }_{3},{\varepsilon }_{2} < I < \sum _{k\mathrm{=1}}^{n}{\mathop{S}\limits^{\breve{}}}_{k}^{0}\\  &  & -n{\varepsilon }_{1}-{\varepsilon }_{3},n{\varepsilon }_{1}+{\varepsilon }_{2} < \sum _{k\mathrm{=1}}^{n}{S}_{k}+I < \sum _{k\mathrm{=1}}^{n}{\mathop{S}\limits^{\breve{}}}_{k}^{0}-{\varepsilon }_{3}\},\end{array}$$

in which *ε*_1_, *ε*_2_, *ε*_3_ are sufficiently small constants.

That is to say the disease will proceed and can not spread to whole susceptible population or go to extinct.

We have illustrated our theoretical results with computer simulations. Finally, this paper is only a first step in introducing switching regime into an epidemic model. In future investigations, we plan to introduce white and color noises into more realistic epidemic models.

Some interesting topics deserve further consideration. On the one hand, one may propose some more realistic but complex models, such as considering the effects of impulsive perturbations on system (1.8). On the other hand, it is necessary to reveal that the methods used in this paper can be also applied to investigate other interesting epidemic models. We leave these as our future work.

## Electronic supplementary material

Supplementary information
