# Supplementary material for: Dynamics of hybrid switching DS-I-A epidemic model
Source: Sci Rep. 2017 Sep 26;7:12332. doi: 10.1038/s41598-017-11901-x (PMC5615109; doi:10.1038/s41598-017-11901-x)
Supplement: Supplementary file 1 — Supplementary information [file 41598_2017_11901_MOESM1_ESM.pdf]

Dynamics of hybrid switching DS-I-A epidemic model

Songnan Liu[1] Daqing Jiang[1,2,*] Xiaojie Xu[1] Tasawar Hayat[2,3] Bashir Ahmad[2]

[1] College of Science, China University of Petroleum (East China), Qingdao 266580, China.

[2] Nonlinear Analysis and Applied Mathematics (NAAM)-Research Group, Department of Mathematics, King Abdulaziz University, Jeddah, Saudi Arabia.

[3] Department of Mathematics, Quaid-I-Azam University 45320, Islamabad 44000, Pakistan.

[*] Correspondence to Daqing Jiang (daqingjiang2010@hotmail.com).

A supplementary information

In this section, we will test our theory conclusion by the Milstein's Higher Order Method in [38]. We first consider the case of system (1.8) with constant coefficients when $n = 2$. Let $(r(t))_{t0}$ be a right-continuous Markov chain taking values in $\mathcal{M} = 1, 2$ with the generator

$$\Gamma = \begin{pmatrix} -0.7 & 0.7 \\ 0.3 & -0.3 \end{pmatrix},$$

By solving the linear equation (2.3) we obtain the unique stationary (probability) distribution

$$\pi = (\pi_1, \pi_2) = (0.3, 0.7).$$

A.1 Extinction

We can get the SDE (1.8) as the result of the following forms switching from one to another according to the movement of the Markovian chain:

$$\begin{cases} dS_k(t) &= [\mu(1)(S_k^0(1) - S_k(t)) - \frac{\beta(1)\alpha_k(1)S_k(t)I(t)}{N(t)}]dt + \sigma_k(1)\frac{S_k(t)I(t)}{N(t)}dB_k(t), \quad 1 \le k \le 2, \\ dI(t) &= [\sum_{k=1}^{n}\frac{\beta(1)\alpha_k(1)S_k(t)I(t)}{N(t)} - (\mu(1) + \gamma(1))I(t)]dt + \sum_{k=1}^{n}\sigma_k(1)\frac{S_k(t)I(t)}{N(t)}dB_k(t), \end{cases}$$

where $\alpha_1(1) = 0.2, \alpha_2(1) = 0.1, S_1^0(1) = 1.5, S_2^0(1) = 1.4, \mu(1) = 0.014, \gamma(1) = 0.3, \beta(1) = 0.3.$

$$\begin{cases} dS_k(t) &= [\mu(2)(S_k^0(2) - S_k(t)) - \frac{\beta(2)\alpha_k(2)S_k(t)I(t)}{N(t)}]dt + \sigma_k(2)\frac{S_k(t)I(t)}{N(t)}dB_k(t), \quad 1 \le k \le 2, \\ dI(t) &= [\sum_{k=1}^{n}\frac{\beta(2)\alpha_k(2)S_k(t)I(t)}{N(t)} - (\mu(2) + \gamma(2))I(t)]dt + \sum_{k=1}^{n}\sigma_k(2)\frac{S_k(t)I(t)}{N(t)}dB_k(t), \end{cases}$$

in which $\alpha_1(2) = 0.21, \alpha_2(2) = 0.18, S_1^0(2) = 1.4, S_2^0(2) = 1.2, \mu(2) = 0.015, \gamma(2) = 0.301, \beta(2) = 0.31.$

Example A.1 *To illustrate the extinction case of system, when condition (1) of Theorem 4.1 is satisfied. If we choose $\sigma_i = (0.4, 0.2),\ i = 1, 2,$ thus $\sigma_k^2(l) < \beta(l)\alpha_k(l),,\ k = 1, 2.$ That is to say the system with strong noise.*

Then the solution $(S_1(t), S_2(t), I(t))$ of system (1.8) with any initial value $(S_1(0), S_2(0), I(0)) = (0.8, 0.8, 2) \in R_+^3$, obeys

$$\bar{R}_0^* := \frac{\sum_{k=1}^{n}\sum_{l=1}^{L}\pi_l\frac{\beta^2(l)\alpha_k^2(l)}{2\sigma_k^2(l)}}{\sum_{l=1}^{L}\pi_l(\mu(l) + \gamma(l))} \approx 0.4572 < 1.$$

Hence, the extinction condition (1) of Theorem 4.1 is satisfied. Therefore by condition of Theorems 4.1, we can obtain that $I(t)$ will tend to zero exponentially with probability one. Using the Milstein's Higher Order Method, we give the simulations shown in Fig.1 and Fig.2 to support our results.

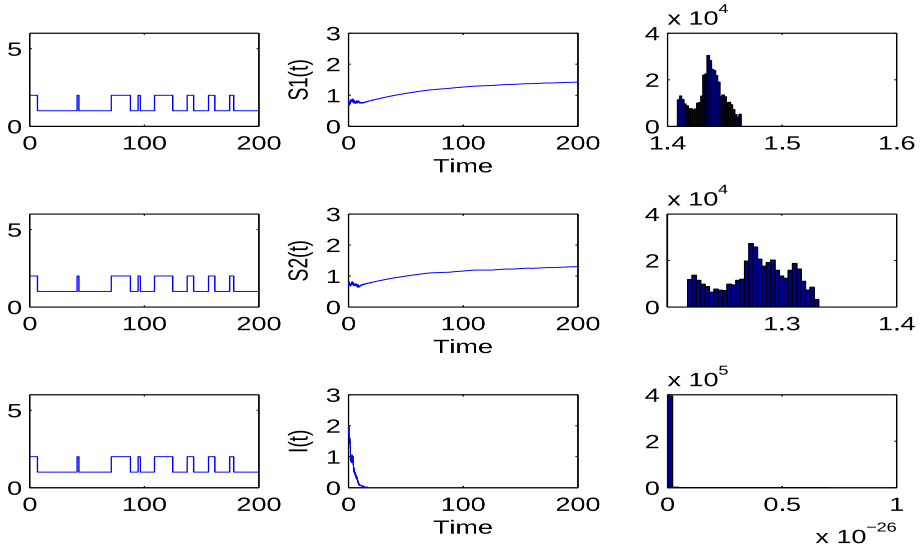

Figure 1: $I(t)$ will tend to zero exponentially with probability one. The pictures on the left are Markovian chain. The pictures on the right are the density functions of system (1.8) for $l \in \mathcal{M} = \{1, 2\}$. We employ the Milstein's Higher Order Method with initial value $(S_1(0), S_2(0), I(0)) = (0.8, 0.8, 2)$.

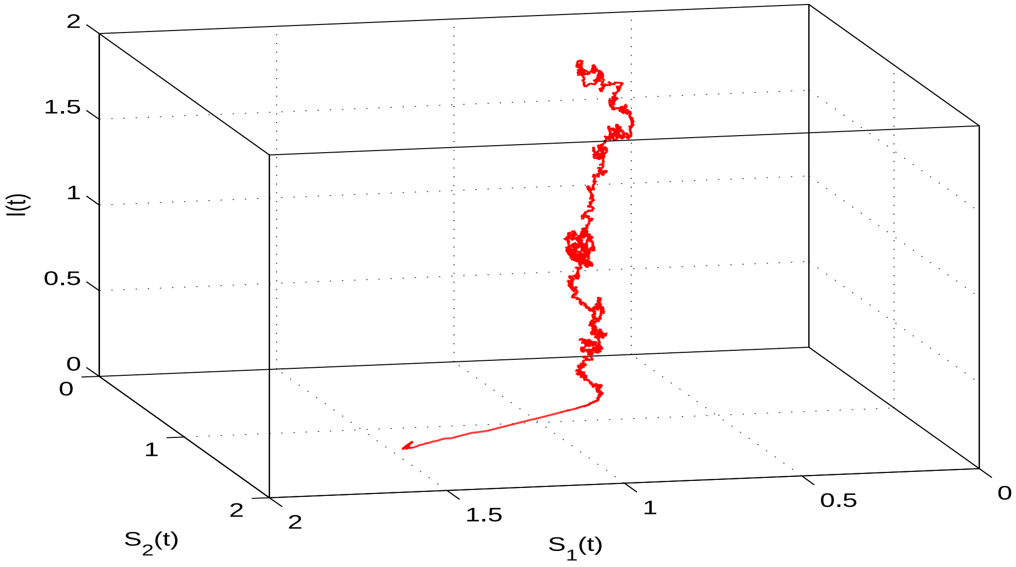

Figure 2: Computer simulation of a single path of $(S_1(t), S_2(t), I(t))$ for the SDE model (1.8) with intial condition $(0.8, 0.8, 2)$.

Example A.2 *Furthermore we discussed the condition (2) of Theorem 4.1 that is* $\sigma_k^2(l) < \beta(l)\alpha_k(l)$, *where* $k = 1, 2, \ldots, n$, $\forall l \in \mathcal{M}$. *If we choose* $\sigma_i = (0.2, 0.15),\ i = 1, 2$. *That is to say the system with with weak noise.*

Then the solution $(S_1(t), S_2(t), I(t))$ *of system* (1.8) *with any initial value* $(S_1(0), S_2(0), I(0)) = (0.8, 0.8, 2) \in R_+^3$, *obeys*

$$\bar{R}_0^* := \frac{\sum_{k=1}^{n}\sum_{l=1}^{L}\pi_l\beta(l)\alpha_k(l)}{\sum_{l=1}^{L}\pi_l\Big(\mu(l) + \gamma(l) + \sum_{k=1}^{n}\frac{\sigma_k^2(l)}{2}\Big)} \approx 0.3273 < 1.$$

Therefore by condition of Theorems 4.1, we can obtain that $I(t)$ *will tend to zero exponentially with probability one.*

Using the Milstein's Higher Order Method, we give the simulations shown in Fig.3 and Fig.4 to support our results.

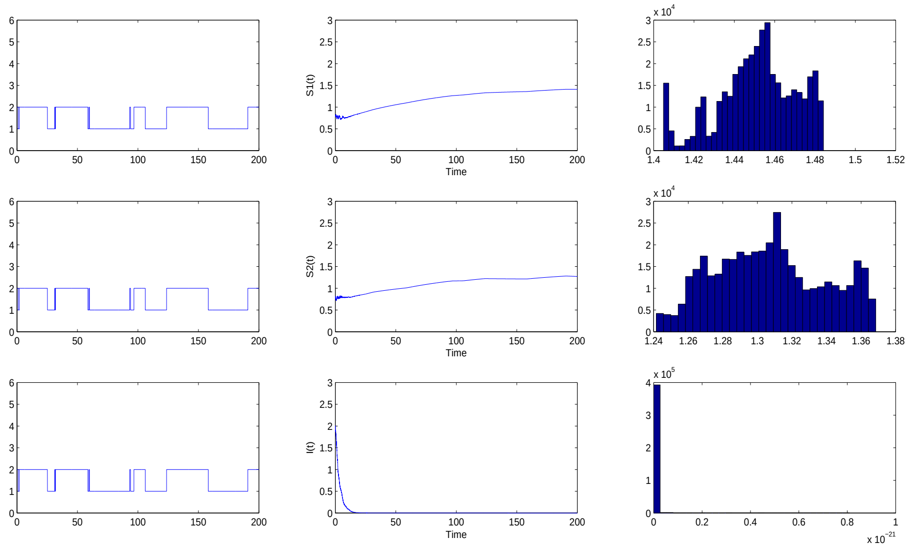

Figure 3: $I(t)$ will tend to zero exponentially with probability one. The pictures on the left are Markovian chain. The pictures on the right are the density functions of system (1.8) for $l \in \mathcal{M} = \{1, 2\}$. We employ the Milstein's Higher Order Method with initial value $(S_1(0), S_2(0), I(0)) = (0.8, 0.8, 2)$.

Remark A.1 *Example 7.1 discussed the condition that the stochastic perturbation are strong, the disease will die out. And Example 7.2 discussed the condition that the stochastic perturbation are weak, the disease will die out under certain conditions.*

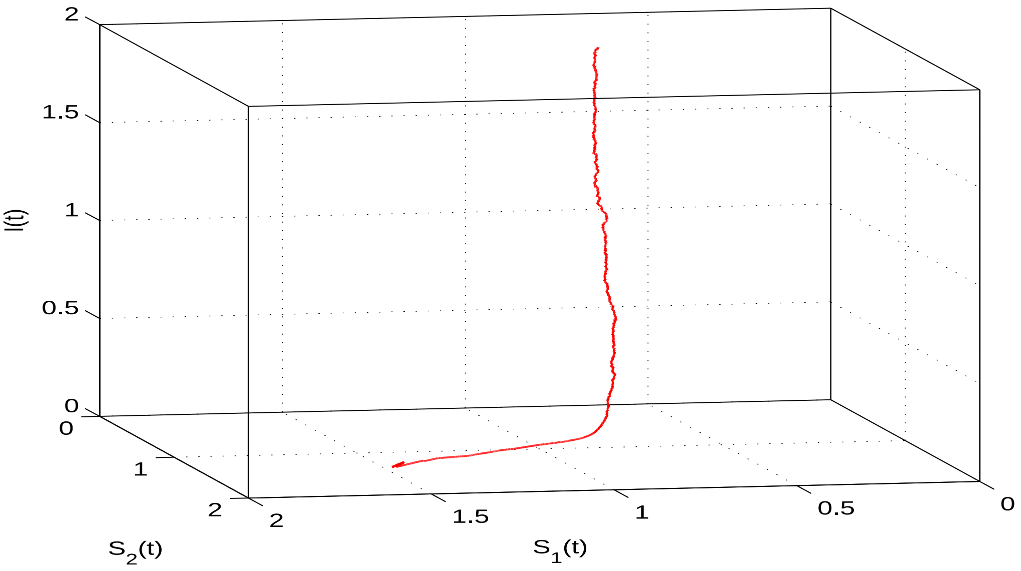

Figure 4: Computer simulation of a single path of $(S_1(t), S_2(t), I(t))$ for the SDE model (1.8) with initial condition $(0.8, 0.8, 2)$.

A.2 Persistence

Example A.3 *To illustrate the Persistence case of system, that is the condition which the disease will proceed. We can get the SDE* (1.8) *as the result of the following forms switching from one to another according to the movement of the Markovian chain:*

$$\begin{cases} dS_k(t) &= [\mu(1)(S_k^0(1) - S_k(t)) - \frac{\beta(1)\alpha_k(1)S_k(t)I(t)}{N(t)}]dt + \sigma_k(1)\frac{S_k(t)I(t)}{N(t)}dB_k(t), \quad 1 \le k \le 2, \\ dI(t) &= [\sum_{k=1}^{n}\frac{\beta(1)\alpha_k(1)S_k(t)I(t)}{N(t)} - (\mu(1)+\gamma(1))I(t)]dt + \sum_{k=1}^{n}\sigma_k(1)\frac{S_k(t)I(t)}{N(t)}dB_k(t), \end{cases}$$

where $\alpha_1(1) = 1.2, \alpha_2(1) = 1, S_1^0(1) = 1.5, S_2^0(1) = 1.4, \mu(1) = 1.2, \gamma(1) = 1.4, \beta(1) = 3$ *and* $\sigma_i(1) = 0.2, i = 1, 2.$

$$\begin{cases} dS_k(t) &= [\mu(2)(S_k^0(2) - S_k(t)) - \frac{\beta(2)\alpha_k(2)S_k(t)I(t)}{N(t)}]dt + \sigma_k(2)\frac{S_k(t)I(t)}{N(t)}dB_k(t), \quad 1 \le k \le 2, \\ dI(t) &= [\sum_{k=1}^{n}\frac{\beta(2)\alpha_k(2)S_k(t)I(t)}{N(t)} - (\mu(2)+\gamma(2))I(t)]dt + \sum_{k=1}^{n}\sigma_k(2)\frac{S_k(t)I(t)}{N(t)}dB_k(t), \end{cases}$$

in which $\alpha_1(2) = 1, \alpha_2(2) = 0.8, S_1^0(2) = 1.4, S_2^0(2) = 1.2, \mu(2) = 1.4, \gamma(2) = 1.2, \beta(2) = 3.2$ *and* $\sigma_i(2) = 0.4, i = 1, 2.$

Then the solution $(S_1(t), S_2(t), I(t))$ *of system* (1.8) *with any initial value* $(S_1(0), S_2(0), I(0)) =$

$(0.8, 0.8, 2) \in R^3_+$, *obeys*

$$R_0^* := \sum_{k=1}^{n} \frac{\Big[\sum_{l=1}^{L}\pi_l(\mu^2(l)\beta(l)\alpha_k(l)S_k^0(l))^{\frac{1}{3}}\Big]^3}{\sum_{l=1}^{L}\pi_l(\mu(l)+\frac{\sigma_k^2(l)}{2})\sum_{l=1}^{L}\pi_l(\mu(l)+\gamma(l)+\sum_{l=1}^{n}\frac{\sigma_k^2(l)}{2})\sum_{l=1}^{L}\pi_l(\mu(l)\sum_{k=1}^{n}S_k^0(l))} \approx 1.0576 > 1.$$

Therefore by condition of Theorems 6.1, as the result of Markovian switching, the solution $(S_1(t), S_2(t), I(t))$ *of system* (1.8) *with any initial value* $(S_1(0), S_2(0), I(0)) = (0.8, 0.8, 2) \in \mathbb{R}^3_+$. *System (1.8) is positive recurrent. That is to say, the disease will proceed.*

Using the Milstein's Higher Order Method, we give the simulations shown in Fig.5 and Fig.6 to support our results.

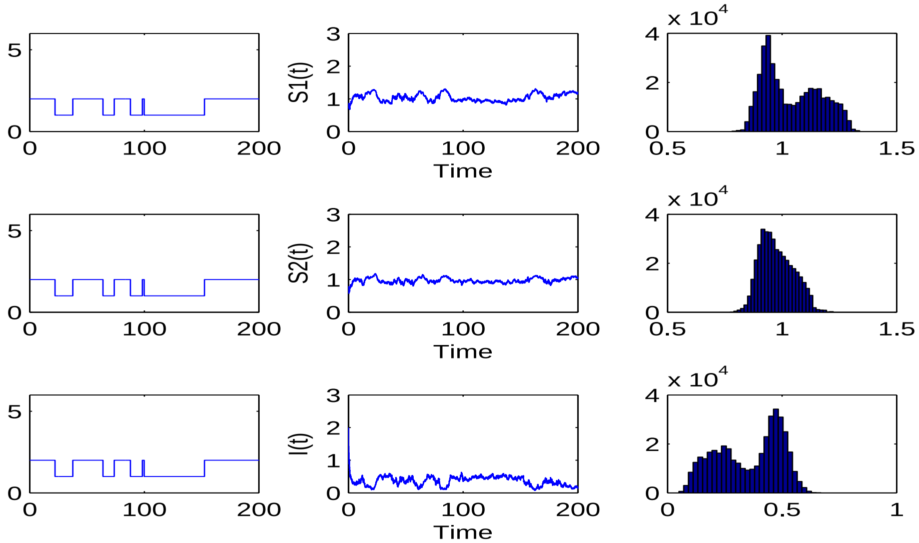

Figure 5: $(S_1(t), S_2(t), I(t))$ is persistent. The pictures on the left are Markovian chain. The pictures on the right are the density functions of system (1.8) for $l \in \mathcal{M} = \{1, 2\}$. We employ the Milstein's Higher Order Method with initial value $(S_1(0), S_2(0), I(0)) = (0.8, 0.8, 2)$.

Then combine Example 7.1 and Example 7.3, if the system (1.8) switching from the Case 1 in Example 7.1 to Case 1 in Example 7.1 to the movement of the Markovian chain. We will discuss the dynamics of system (1.8).

Example A.4 *We can get the SDE* (1.8) *as the result of the following forms switching from one to another according to the movement of the Markovian chain:*

$$\begin{cases} dS_k(t) &= [\mu(1)(S_k^0(1) - S_k(t)) - \frac{\beta(1)\alpha_k(1)S_k(t)I(t)}{N(t)}]dt + \sigma_k(1)\frac{S_k(t)I(t)}{N(t)}dB_k(t), \quad 1 \le k \le 2, \\ dI(t) &= [\sum_{k=1}^{n}\frac{\beta(1)\alpha_k(1)S_k(t)I(t)}{N(t)} - (\mu(1)+\gamma(1))I(t)]dt + \sum_{k=1}^{n}\sigma_k(1)\frac{S_k(t)I(t)}{N(t)}dB_k(t), \end{cases}$$

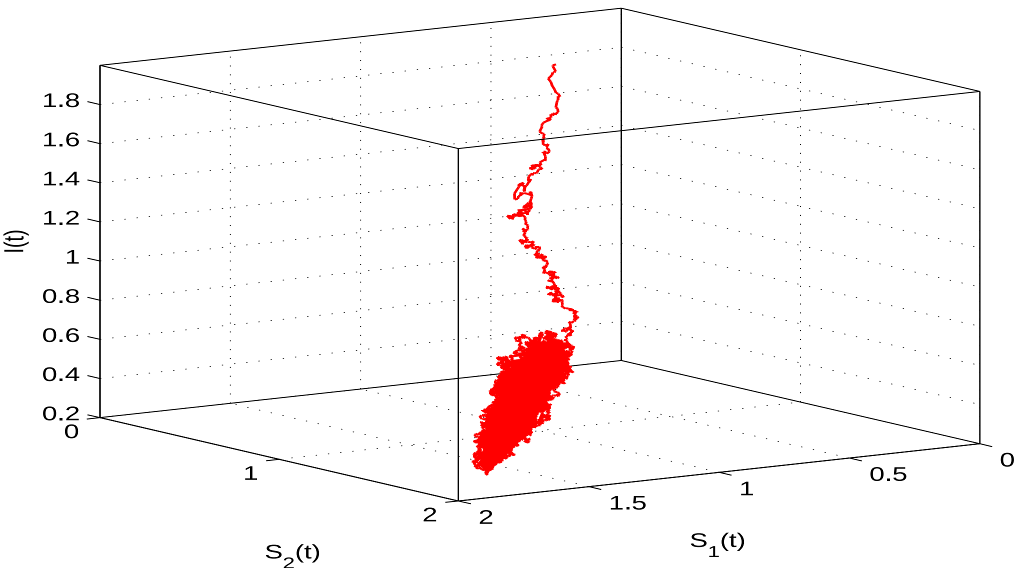

Figure 6: Computer simulation of a single path of $(S_1(t), S_2(t), I(t))$ for the SDE model (1.8) with initial condition $(0.8, 0.8, 2)$.

where $\alpha_1(1) = 1.2, \alpha_2(1) = 1, S_1^0(1) = 1.5, S_2^0(1) = 1.4, \mu(1) = 1.2, \gamma(1) = 1.4, \beta(1) = 3$ *and* $\sigma_i(1) = 0.2, i = 1, 2.$

$$\begin{cases} dS_k(t) &= [\mu(1)(S_k^0(1) - S_k(t)) - \frac{\beta(1)\alpha_k(1)S_k(t)I(t)}{N(t)}]dt + \sigma_k(1)\frac{S_k(t)I(t)}{N(t)}dB_k(t), \quad 1 \le k \le 2, \\ dI(t) &= [\sum_{k=1}^{n} \frac{\beta(1)\alpha_k(1)S_k(t)I(t)}{N(t)} - (\mu(1) + \gamma(1))I(t)]dt + \sum_{k=1}^{n} \sigma_k(1)\frac{S_k(t)I(t)}{N(t)}dB_k(t), \end{cases}$$

where $\alpha_1(2) = 0.2, \alpha_2(2) = 0.1, S_1^0(2) = 1.5, S_2^0(2) = 1.4, \mu(2) = 0.014, \gamma(2) = 0.3, \beta(2) = 0.3$ *and* $\sigma_i(2) = 0.4, i = 1, 2.$

Then the solution $(S_1(t), S_2(t), I(t))$ *of system* (1.8) *with any initial value* $(S_1(0), S_2(0), I(0)) = (0.8, 0.8, 2) \in R_+^3$*, obeys*

$$R_0^* := \sum_{k=1}^{n} \frac{\left[\sum_{l=1}^{L} \pi_l (\mu^2(l)\beta(l)\alpha_k(l)S_k^0(l))^{\frac{1}{3}}\right]^3}{\sum_{l=1}^{L} \pi_l(\mu(l) + \frac{\sigma_k^2(l)}{2})\sum_{l=1}^{L} \pi_l(\mu(l) + \gamma(l) + \sum_{l=1}^{n} \frac{\sigma_k^2(l)}{2})\sum_{l=1}^{L} \pi_l(\mu(l)\sum_{k=1}^{n} S_k^0(l))} \approx 1.3306 > 1.$$

Therefore by condition of Theorems 6.1, as the result of Markovian switching, the solution $(S_1(t), S_2(t), I(t))$ *of system* (1.8) *with any initial value* $(S_1(0), S_2(0), I(0)) = (0.8, 0.8, 2) \in \mathbb{R}_+^3$*. System* (1.8) *is persistent. That is to say, the disease will proceed.*

Using the Milstein's Higher Order Method, we give the simulations shown in Fig.7 and Fig.8 to support our results.

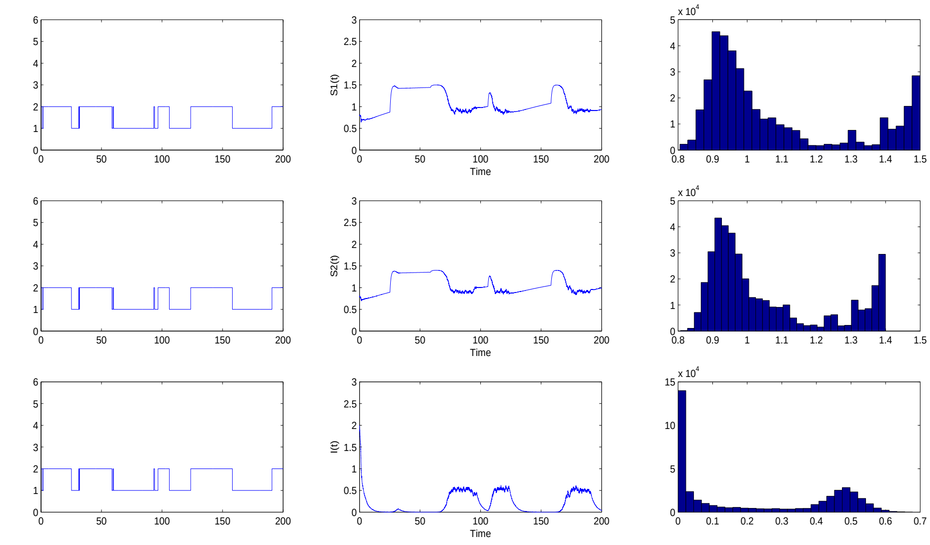

Figure 7: $(S_1(t), S_2(t), I(t))$ is persistent. The pictures on the left are Markovian chain. The pictures on the right are the density functions of system (1.8) for $l \in \mathcal{M} = \{1, 2\}$. We employ the Milstein's Higher Order Method with initial value $(S_1(0), S_2(0), I(0)) = (0.8, 0.8, 2)$.

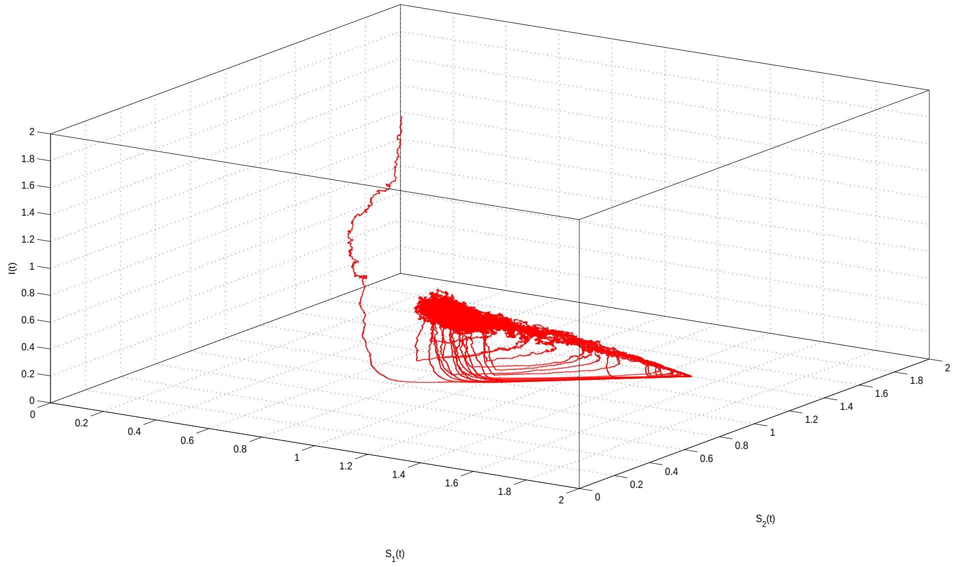

Figure 8: Computer simulation of a single path of $(S_1(t), S_2(t), I(t))$ for the SDE model (1.8) with initial condition $(0.8, 0.8, 2)$.

Remark A.2 *According to* (1.4)*, we can obtain* $R_0 \approx 1.2732 > 1$*, when* $l = 1$*. That is to say the corresponding deterministic of* (1.8) *is persistent. When* $l = 2$*, then the* $R_0 \approx 0.1449 < 1$*. That is to say the corresponding deterministic of* (1.8) *will die out. Thus Example 7.4 tells us that the disease proceeds when it switches between the proceed condition and the end condition, if the generator of the Markov chain* γ_{ij} *be*

$$\Gamma = \begin{pmatrix} -0.7 & 0.7 \\ 0.3 & -0.3 \end{pmatrix}.$$
